# Examination of hydro climatic trend and drought analysis for climate resilience in the eastern escarpment of Abaya Chamo sub-basin, Rift Valley Lake Basin, Ethiopia

**DOI:** 10.1371/journal.pone.0354913

**Published:** 2026-08-06

**Authors:** Muse Wldmchel Shomre, Dawit Yohannes Meskele, Yishak Beyene

**Affiliations:** 1 Wachemo University, Hydraulic and Water Resources Engineering Department, Hosaena, Ethiopia; 2 Wachemo University, Hydraulic and Water Resources Engineering Department, Hosaena, Ethiopia; 3 Wachemo University, College of Agricultural Science, Hosaena, Ethiopia; Euro-Mediterranean Center for Climate Change: Fondazione Centro Euro-Mediterraneo sui Cambiamenti Climatici, ITALY

## Abstract

A robust assessment of hydroclimatic variability is critical for strengthening drought resilience and optimizing water resource management in climate-sensitive and data-limited regions. This study provides one of the first comprehensive integrated hydroclimatic analyses of the eastern escarpment of the Abaya-Chamo sub-basin, evaluating multi-decadal trends in rainfall, temperature, streamflow, and drought through the Mann-Kendall trend test, Indicators of Hydrologic Alteration (IHA), and drought indices derived using DrinC software. Rainfall trends were largely insignificant across five monitoring stations, except at Hagereselam station, which exhibited an upward trend. Maximum monthly temperatures increased significantly at all stations except Dilla, while minimum temperatures rose at Fisehagenet, Yirgachefe, and Hagereselam. Streamflow analysis at the Tore gauging station revealed elevated low flows, reduced peak flows, slower recession rates, slightly decreased winter discharges, more frequent pulse events, and a reversal in flow direction. Drought assessment using SPI-6 and RDI_st_-6 indicated increased frequency and severity in recent decades, with Arbaminch, Yirgachefe, Dilla, and Hagereselam being particularly affected. The most severe drought levels were recorded at Yirgachefe (index score −3.31) and Arbaminch (index score −3.27) stations. Short-term indices (SPI-6 and RDI_st_-6) were more effective than 12-month indices in capturing recurring drought episodes, highlighting the importance of multi-scale drought monitoring. By linking meteorological drought with hydrological regime alterations, this integrated framework provided a comprehensive understanding of drought propagation and hydroclimatic interactions. These findings provide essential guidance for adaptive water governance and region-specific drought mitigation in a region increasingly vulnerable to climate change and broader climate drivers such as El Niño-Southern Oscillation (ENSO), underscoring the urgency of timely interventions in response to ongoing climatic variability.

## Introduction

Numerous scientific studies have shown that climate change has adverse impacts on socioeconomic development, although the severity of its effects varies across countries and hydrological catchments [1]. Globally, greenhouse gas emissions and atmospheric CO_2_ concentrations have increased, causing the average global temperature to rise by 0.74°C compared to pre-industrial levels [2]. In large parts of Africa, near-surface temperatures have risen at least 0.5°C for the past 50–100 years [3]. The Intergovernmental Panel on Climate Change (IPCC) predicts that by 2100, the average global temperature will increase by 1.4°C to 5.8°C relative to 1990 levels, while mean annual precipitation may vary by up to 20% [4]. The IPCC conducted studies on numerous physical and biological systems and concluded that variability in temperature, precipitation intensity, and extreme weather frequency could have a long-term impact on global warming [5].

Currently, climatic variability poses significant challenges to water sustainability, particularly affecting agricultural production in regions such as southern Ethiopia [6], where crop yields are highly sensitive to variations in temperature and rainfall [7]. A continuous decline in rainfall trends at meteorological stations can lead to water resource deficits and drought, both posing serious ecological hazards [8]. Studies of regional and global climatic variability rely primarily on reliable climate data, which are critical for hydrological modeling, climate forecasting, and water resource management [9]. Climate trend detection provides clear indicators of global warming and forms a basis for water resource management at regional and sub-basin levels [10]. Ethiopia, despite being the water tower of East Africa, faces substantial water-related stress due to natural disasters such as drought and flooding, which are directly linked to climatic variability [11].

Drought is characterized by an extended period of below-average precipitation, which may last a season, a year, or for multiple years [12], gradually affecting large populations and often escaping global attention [13]. Drought has a significant negative impact on agriculture, human health, and the economy in Africa, particularly in sub-Saharan countries, posing a serious threat to livelihoods and overall human well-being [14,15]. Drought can be classified as agricultural, hydrological, meteorological, or socioeconomic depending on the system affected, while its intensity, duration, and spatial extent determine its severity [16,17]. Consequently, hydro-meteorological trends have been widely investigated using both parametric and nonparametric approaches to better understand climatic variability and its impacts [18,19].

Non-parametric trend tests, such as the Mann-Kendall test, are widely used because they do not require assumptions about data distributions, whereas parametric tests rely on distribution assumptions [20,21]. Different studies have reported varying conclusions depending on catchment characteristics and methodological approaches [22]. Mann-Kendall trend tests, Sen’s slope estimators, and Indicators of Hydrologic Alteration (IHA) have been used to analyze climatic and hydrological trends across diverse catchments [23,24]. The Mann-Kendall trend test is particularly powerful for identifying trends in time series data, including temperature and rainfall [25,26]. Studies using the Mann-Kendall trend test have demonstrated greater confidence than linear regression in assessing temperature trends in Papua, Indonesia [27]. In East Java, rainfall at several meteorological stations showed significant declines [28]. In Ethiopia, decreasing rainfall trends have been reported in the southwestern, southeastern, and eastern regions, while northern and central areas show no significant temporal changes [29].

Annual precipitation variability in the Woleka sub-basin of northern Ethiopia demonstrated both intra-annual and inter-annual fluctuations attributable to climate variability [30]. Regional studies indicate decreasing rainfall trend in the southwest highlands and along the Sudanese border, while minor increase occurred in southern and southeastern lowlands [31]. In contrast, western Ethiopia shows increasing rainfall trends with pronounced regional and temporal variability [32]. Most gauging stations in the Upper Blue Nile basin exhibited a significant seasonal and annual runoff increase [33]. Understanding climate change and variability is crucial for maintaining water balance and effective resource protection [24]. Despite numerous studies on climatic trends and drought across different Ethiopian river basins [32,34–37], significant knowledge gaps remain in the eastern escarpment of the Abaya Chamo sub-basin.

Previous research has predominately focused on rainfall and temperature trend at national or large basin scales, often overlooking localized streamflow changes and their associated environmental implications [37,38]. Furthermore, the application of Indicators of Hydrologic Alteration to evaluate detailed flow regime characteristics in Ethiopian river systems has been limited [39,40]. Many drought studies rely on single indices, whereas integrating multiple indices such as the Standardized precipitation Index(SPI) and the Reconnaissance Drought Index (RDI) provide a more comprehensive understanding of drought severity, duration, and frequency [17,41]. However, SPI and RDI are rarely incorporated into hydrologic regime metrics such as IHA, limiting understanding of how meteorological droughts propagate into hydrological drought [16].This limitation is particularly evident in data-scarce sub-basins such as the Abaya-Chamo sub-basin, where integrated hydroclimatic analyses remain limited.

Hydrological drought analysis in Ethiopia seldom employs ecologically relevant flow regime metrics, instead focusing on meteorological or simplified streamflow indices. This limits understanding of drought propagation and ecosystem impacts [16,42,43]. To address these gaps, this study integrated drought derived from DrinC software with IHA metrics, based on multi- decadal meteorological and hydrological datasets. This model-independent framework enables robust assessment of climate-hydrology linkages in a data scarce region. By providing regionally focused and methodologically integrated analysis, this study contributes new insights into hydroclimatic variability and streamflow alteration in the Abaya- Chamo sub-basin, essential for water resource management, ecological conservation, and climate adaptation planning. Also, high quality temporal and spatial data, combined with multiple analytical tools, are critical for reducing existing knowledge gaps and facilitating coherent, evidence -based decision making [44].

Proper examination of sub-basin constraints is critical for future water resource management, planning, and policymaking, particularly for surface water systems that are highly dependent on rainfall [45]. Changes in measurement techniques, observational practices, and shift in station locations are common non-climatic sources of data inhomogeneity [10]. Because most studies on climatic trends are conducted at regional or national scales, which may not capture sub basin-level characteristics due to the wide range of climatic variability, it is essential to use accurate observational data that have been properly validated and tested for inhomogeneity when conducting hydroclimatic trend analyses [46,47]. Drought is a natural hazard, and its characterization across watersheds is commonly achieved using various indices [48,49].

A variety of variables are used to describe different types of drought indicators. For example, meteorological indicators focus on weather variables such as precipitation, temperature, and evapotranspiration; hydrological indicators reflect water availability in rivers, lakes, and groundwater; and soil moisture indicators are crucial for agriculture and ecosystem functioning [49,50]. Multiple indices simplify complex data, facilitating comparison of drought conditions across locations and time [51]. Numerous drought indices, including the Reconnaissance Drought Index (RDI), Standardized Precipitation Index (SPI), Standardized Precipitation Evapotranspiration Index (SPEI), Decile Index, Palmer Drought Severity Index (PDSI), and Streamflow Drought Index (SDI), have been developed based on varying inputs [50,52–54]. To forecast drought conditions, many studies employ combinations of multiple indices [14,16,50].

Comprehensive assessments using multiple indices provide benchmarks for evidence-based policymaking and facilitate the development of locally tailored strategies to reduce drought-related hazards [38,50,52,55,56]. Various studies have identified the SPI and RDI as key climatic indices for detecting potential climatic variability among numerous available drought indices [57]. For example, SPI have been widely applied to assess rainfall deficits across multiple timescales. In Tegal city, Central Java,SPI analysis revealed the most severe drought event within the study period in 2015 [58]. In the Upper Niger Basin, SPI was identified as the most suitable meteorological drought index [59], reinforces robustness. However, the literature reviews consistently emphasizes that no single drought index is universally sufficient for comprehensive drought analysis. Instead, the combined application of multiple indices has been shown to improve the accuracy and reliability of drought characterization [53].

Drought variability in tropical is increasingly driven by the interaction of large-scale climate modes rather than isolated factors. In specifically, the combined influences of ENSO, ENSO Modoki, and volcanic forcing alters global circulations leading to significant shifts in rainfall patterns across Africa. Understanding these coupled climate drivers is therefore essential for improving drought and water resource management under a changing climatic conditions [60–63]. In Ethiopia, numerous drought-related studies have been conducted, particularly in the northern and eastern regions of the country, providing important insights into drought characteristics and impacts [53,54]. The eastern escarpment of the Abaya Chamo sub-basin encompasses an important ecological and tourism area, while stations such as Arbaminch, Yirgachefe, Dilla, Hagereselam, and Fisehagenet experience substantial year-round climatic variability, which affects agriculture and small-scale irrigation. However, the sub-basin suffers from a lack of detailed hydroclimatic drought data, complicating effective drought monitoring and management. These finding aims to bridge knowledge gaps and inform targeted, location-specific interventions at the catchment scale, moving beyond conventional top-down policy approaches to foster timely and locally relevant strategies for water resource management and drought mitigation.

## Materials and methods

### Description of the study area

**Fig 1:**
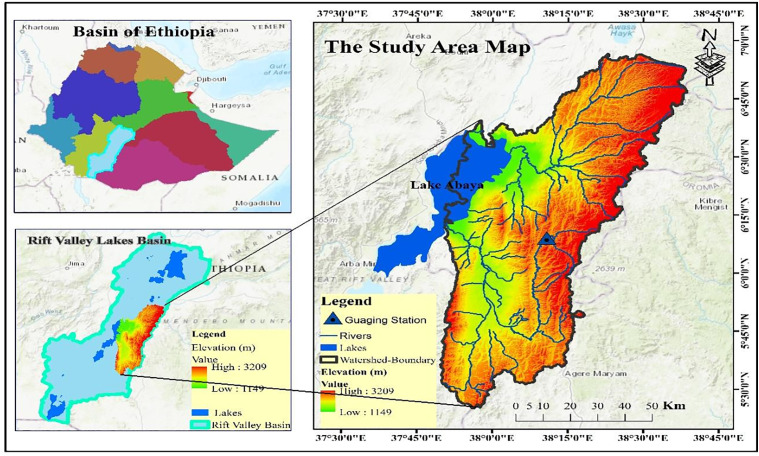
Geographical location of the study area. The main map displays the topography, rivers, lakes, watershed boundary, and elevation variation of the sub-basin. The elevation and watershed boundary data were obtained from the USGS watershed boundary dataset (https://www.usgs.gov/). All geospatial layers were processed and rendered using open-source GIS software in compliance with open-access licensing.

The research was conducted in the eastern escarpment of the Abaya-Chamo (AC) sub-basin, located within the Rift Valley Lake basin of Ethiopia (Fig 1). The sub-basin lies to the south of Lake Abaya and extends approximately between 5°25′ and 6°18′ North latitude and 37°50′ to 38°20′ East longitude. The eastern escarpment of the AC sub-basin exhibits considerable climatic heterogeneity, ranging from semi-arid conditions in the lowlands to humid conditions in the highlands of the catchment [64]. The watershed experiences pronounced seasonal climatic variability, with no stable climatic conditions throughout the year. Annual precipitation varies considerably, ranging from approximately 665 mm in the lowland areas to about 1800mm in the highland regions [65]. Mean annual temperatures across the basin range from 9 °C in the highlands to 24 °C in the lowlands [65]. Rainfall in the basin follows a bimodal pattern, with peak rainfall occurring during April to May (the first rain season) and September to October (the second rainy season) [66]. Agricultural land constitutes the dominant land use land cover in the basin and has been rapidly expanding due to population growth and intensified agricultural activities. The elevation of the basin ranges from approximately 1,100–3,045 m above sea level.

### Data sources

The main data used in this study consisted of meteorological and hydrological records collected from various stations over different time periods, as shown in Table 1. Based on data completeness and reliability, six meteorological stations were selected from study area and those data were acquired from Ethiopian National Meteorological Agency (ENMA). Two hydrological stations (Tore and Yirgachefe) were obtained from the Ministry of Water and Energy (MoWE). Several datasets contained distortions and missing values, because many stations were located near agricultural field. To ensure data quality, advanced gap-filling techniques were applied. Missing temperature data (maximum and minimum) were filled by using spatial interpolation methods, particularly Inverse Distance Weighting (IDW), which is appropriate due to the smooth spatial variation of temperature. Precipitation data, were more variable in space and time, completed using multiple imputation techniques. For the hydrological data, missing streamflow records were reconstructed using regression equations developed based on data from the Tore and Yirgachefe gauging stations.

**Table 1:** List of the selected meteorological and hydrological stations.

Station	Latitude	Longitude	Elevation	Period of study	Number of years spanned
Arbaminch	6.1	37.6	1207	1987-2019	33
Yirgachefe	6.2	38.2	1856	1987-2019	33
Hageremariam	5.7	38.2	1861	1987-2019	33
Fisegenet	6.1	38.2	2240	1987-2019	33
Hagereselam	6.48	38.52	2759	1990-2021	33
Dilla	6.41	38.01	1711	1990-2021	32
Tore gauging	5.93	38.16	1715	1990-2013	24

### Climatic variables and trend analysis

In this study, XLSTAT (version 2020.5) was used to detect trend parameters, including the Mann-Kendall statistic (S), Sen’s slope, Kendall’s tau, and the standardized Z statistic. In the Mann-Kendall trend test, each observation was compared with subsequent observations. If a data value from a later period was higher than a data value from an earlier period, the statistic S was incremented by 1; if it was lower, S was decremented by 1. The final value of S was obtained by summing all such pairwise comparisons. A large positive value of S indicates an increasing trend, whereas a large negative value indicates a decreasing trend.

Sen’s slope describes the rate of change in the trend per unit time, whereas Kendall’s tau measures the correlation, which reflects the strength of the link between two variables. Kendall’s tau ranges from −1 to +1; a positive value indicates that the ranks of both variables increase concurrently, while a negative value indicates that as the rank of one variable increases, the rank of other decreases. The Z statistic is the standardized test statistic. A trend is considered decreasing when the Z value is negative and the computed probability (P-value) is less than the chosen level of significance. Conversely, if the Z value is positive and the P-value is less than the significance level, the trend is considered increasing.

For each dataset trends were evaluated under either the null or alternative hypothesis. The null hypothesis (H_0_) states that there is no trend in the time series and that the data are independently and identically distributed, whereas the alternative hypothesis (Ha) indicates that the data exhibit a monotonic trend, either increasing or decreasing.

The Mann-Kendall test compares data values in an ordered time series: x1, x2,.., xn, where each value is compared with all subsequent values. For each pair (xi, xj) where j > i, the statistic S incremented by 1 if xj > xi and decremented by 1 if xj < xi. The test statistic S is calculated as follows: -


$$\begin{array}{c} \text{S}=\sum_{\text{i}=\text{1}}^{\text{n}-\text{1}}*\sum_{\text{j}=\text{i}+\text{1}}^{\text{n}}\text{sign}(\text{Tj}-\text{Ti}) \end{array}$$ (1)

$$\begin{array}{c} \text{sign}(\text{Tj}-\text{Ti})=\left\{ \begin{array}{ccc} \text{1} & \text{if} & \text{Tj}-\text{Ti}>\text{0}\\ \text{0} & \text{if} & \text{Tj}-\text{Ti}=\text{0}\\ -\text{1} & \text{if} & \text{Tj}-\text{Ti }<\text{0} \end{array}\right. \end{array}$$ (2)

Where: T_j_ and T_i_ represent the observed values at times j and i, respectively, with j > i.

For n ≥ 10, the statistic S is approximately normally distributed with the mean E(S) and variance (σ2), given by:


$$\begin{array}{c} \text{E}(\text{S})\;=\;\text{0} \end{array}$$ (3)

$$\begin{array}{c} \sigma \text{2}=\frac{\text{n}(\text{n}-\text{1})+(\text{2n}+\text{5})-\Sigma \text{ti}(\text{i})(\text{i}-\text{1})(\text{2i}+\text{5})}{\text{18}} \end{array}$$ (4)

Where: ti denotes the number of tied observations in the i^th^ group. The summation term in the numerator is used only if the data series contains tied values, while the standard test statistic (Zs) is calculated as follows: -


$$\begin{array}{c} Zs=\left\{ \begin{array}{c} @l\text{S}-\frac{\text{1}}{\sqrt{\text{Var}(\text{S})\;}}\text{if S}>\text{0}\\ \text{0}\text{ if S}=\text{0}\\ \text{S}+\frac{\text{1}}{\sqrt{\text{Var}(\text{S})}}\text{ if S}<\text{0} \end{array}\right. \end{array}$$ (5)

The test statistic Zs is used to assess the significance of the trend by testing null hypothesis (H_o_). When |Zs| > Z_α/2_, where α denotes the selected significance level, the null hypothesis is rejected, indicating the presence of a statistically significant trend.

### Sen’s slope estimator

In the present study, the Sen’s slope estimator was applied to quantify the magnitude of trends in temporal datasets. This method is well suited for detecting linear trends, as it is robust to outliers in the data series. Sen’s slope represents the rate of change (magnitude per unit time, e.g., per year) in a time series; positive values indicate an increasing trend, whereas negative values indicate a decreasing trend. This technique provides a robust estimate of trend magnitude even in the presence of non-normal data or missing values, making it particularly suitable for environmental, hydrological, and climate time-series analyses. The magnitude of the trend is estimated as the median of all pairwise slopes (dk), as expressed in equation (6):


$$\begin{array}{c} \text{dk}=\frac{\text{xj}-\text{xi}}{\text{j}-\text{i}} \end{array}$$ (6)

Where: dk is the slope for (1 ≤ i < j ≤ n), x denotes the data value, n is the total number of observations, and i, j is the corresponding time indices.

### Indicators of Hydrologic Alteration (IHA)

The Indicators of Hydrologic Alteration (IHA) is a statistical software tool developed by The Nature Conservancy to assess the extent to which human activities have altered flow regimes. In this study, streamflow data from Tore gauging station, located on the eastern escarpment of the Abaya Chamo sub-basin, were analyzed using the IHA software for over 24-year record (1990–2013). Examining the flow patterns and seasonal variations of the Tore River in the basin is highly advantageous because this river is one of the primary inflows to Lake Abaya and plays a significant role in irrigation, as well as domestic and livestock water supply. The IHA software computes a range of hydrological parameters using either parametric (mean and standard deviation) or non-parametric (median and percentile) statistical approaches. In total, the IHA generates 67 statistical parameters, comprising 33 IHA parameters and 34 Environmental Flow Components (EFCs). Their definitions, grouping, and hydrological significance are presented in Table 2. The IHA parameters are grouped into five categories, each representing a distinct aspect of the flow regime. The mathematical formulations of selected IHA parameters are presented below: -

**Table 2:** Summary of IHA parameters and ecosystem influences.

S. No.	IHA parameter Group	Flow regime characteristics	Hydrologic parameters	Sub-total parameters	Group No
1	Magnitude of monthly water conditions	Magnitude, Timing	Mean or median value for each calendar month	12	One
2	Magnitude and duration of annual extreme water conditions	Magnitude, Duration	Annual minima and maxima based on 1-, 3-, 7-, 30-, and 90-day means; Number of zero-flow days; base flow index (7-day minimum flow/mean flow for the year)	12	Two
3	Timing of annual extreme water conditions	Timing	Julian date of each annual 1-day maximum Julian date of each annual 1-day minimum	2	Three
4	Frequency and duration of high and low flow pulses	Magnitude, Frequency and Duration	Number of low pulses within each water year and the mean or median duration of low pulses (days); number of high pulses within each water year and mean or median duration of high pulses (days)	4	Four
5	Rate and frequency of water condition changes	Rate of change in frequency	Rise rates: mean or median of all positive differences between consecutive daily values; fall rates: mean or median of all negative differences between consecutive daily values; number of hydrologic reversals	3	Five

1. Magnitude of monthly water conditions

For each schedule of a month n = 1, 2...12


$$\begin{array}{c} \text{Mean monthly flow}:\text{ Qm}=\frac{\text{1}}{\text{nm}}*\sum_{\text{i}=\text{1}}^{\text{nm}}\text{qi} \end{array}$$ (7)

$$\begin{array}{c} \text{Median monthly flow}:\text{ Qm}=\text{median }(\text{q1},\text{q2 },\text{q3 },...,\text{ qnm}) \end{array}$$ (8)

1. Magnitude and duration of annual extreme water conditions

For year m, using a sliding window of length K days


$$\begin{array}{c} \text{Annual K}-\text{day minimum flow}:\text{Qk}-\text{day min }={\text{min}}_{\text{1}\leq \text{j}\leq \text{N}-\text{k}+\text{1}}\;(\text{1}/\text{k}*\sum_{\text{i}=\text{j}}^{\text{j}+\text{k}-\text{1}}\text{qi}) \end{array}$$ (9)

$$\begin{array}{c} \text{Annual K}-\text{day maximum flow}:\text{ Qk}-\text{day max }={\text{max}}_{\text{1}\leq \text{j}\leq \text{N}-\text{k}+\text{1}}\;(\text{1}/\text{k}*\sum_{\text{i}=\text{j}}^{\text{j}+\text{k}-\text{1}}\text{qi}) \end{array}$$ (10)

$$\begin{array}{c} \text{Number of zero flow days}:\text{ ZFD}=\sum_{\text{i}=\text{1}}^{\text{N}}\text{II }(\text{qi}=\text{0}) \end{array}$$ (11)

1. Timing of annual extreme water conditions

$$\begin{array}{c} \text{Julian date of maximum flow }(\text{J D max})=\text{arg }{\text{max}}_{\text{1}\leq \text{j}\leq \text{N}-\text{k}+\text{1}}\text{ qi} \end{array}$$ (12)

$$\begin{array}{c} \text{Julian date of minimum flow }(\text{J D min})=\text{arg }{\text{min}}_{\text{1}\leq \text{j}\leq \text{N}-\text{k}+\text{1}}\text{ qi} \end{array}$$ (13)

1. Frequency and duration of high and Low pulse

$$\begin{array}{c} \text{Number of high pulse events }(\text{NHP})=\text{count of continuous interval where qi}>\text{Q75} \end{array}$$ (14)

$$\begin{array}{c} \text{Average duration of high pulse rate }(\text{DHP})=\text{1}/\text{NHP }(\sum_{\text{k}=\text{1}}^{\text{NHP}}\text{dk}) \end{array}$$ (15)

1. Rate and frequency of water condition changes

$$\begin{array}{c} \text{Rise rate }(\text{RR})=\text{1}/{\text{m}}^{+}\;\sum_{\text{i}=\text{2}}^{\text{N}}\text{max}\;(\text{0},{\text{ q}}_{\text{i}}-{\text{ q}}_{\text{i}-\text{1}}) \end{array}$$ (16)

$$\begin{array}{c} \text{Fall rate }(\text{FR})=\text{1}/{\text{m}}^{-}\;\sum_{\text{i}=\text{2}}^{\text{N}}\text{min }(\text{0},{\text{ q}}_{\text{i}}-{\text{ q}}_{\text{i}-\text{1}}) \end{array}$$ (17)

$$\begin{array}{c} \text{Number of reversals }(\text{R})=\sum_{\text{i}=\text{3}}^{\text{N}}\text{II}[\left({\text{ q}}_{\text{i}}-{\text{ q}}_{\text{i}-\text{1}}\right)\;\left({\text{ q}}_{\text{i}-\text{1}}-{\text{ q}}_{\text{i}-\text{2}}\right)<\text{0}] \end{array}$$ (18)

Where: qi is the daily streamflow on day i of month m, nm is the number of days in month m, II is indicator function (equals 1 if the condition is true), dk is duration (in days) of the k^th^ high-pulse event, N is the total number of daily streamflow observations in the year, and m^+^ and m^-^ represent the numbers of rising and falling intervals, respectively.

### Drought assessment and qualitative validation of the exposure risk index

Drought indices were used to quantify the intensity, duration, and magnitude of drought [38,50,55]. These indices are widely applied for drought monitoring and characterization in numerous studies using a variety of analytical tools. In the present study, the Standardized Precipitation Index (SPI) and the Reconnaissance Drought Index (RDI) were calculated using the DrinC (Drought Indices Calculator) software for 6-month and 12-month timescales. For both indices, the reference period for the calculations began in October. The primary computation timescales corresponded to the seasonal (6-month) and annual (12-month) scales. The associated drought severity classes and magnitude ranges are summarized in Table 3.

**Table 3:** Classification of drought and wetness conditions based on SPI and RDI values [56].

SPI and RDI values	Classification	Abbreviation
≥2.00	Extremely wet condition	EXW
1.50 to 1.99	Severely wet condition	SEW
1.00 to 1.49	Moderately wet condition	MOW
−0.99 to 0.99	Near normal condition	NN
−1.00 to −1.49	Moderately dry condition	MOD
−1.50 to −1.99	Severely dry condition	SED
≤−2.00	Extremely dry condition	EXD

#### Standardized precipitation index (SPI).

The Standardized Precipitation Index (SPI) is one of the most widely used drought indicators in scientific research due to its simplicity and flexibility, as it relies solely on precipitation data and can be applied across multiple temporal scales [56,67]. The SPI is calculated as follows [67,68].


$$\begin{array}{c} \text{SPI}=\frac{\text{X}-\mu }{\delta } \end{array}$$ (19)

Where: x is precipitation value, µ is long-term mean precipitation, and δ is standard deviations of long-term precipitation

#### Reconnaissance drought index (RDI).

As an extension to the SPI, the Reconnaissance Drought Index (RDI) accounts for both precipitation and atmospheric evaporative demand, making it a multi-scalar index [69,70]. The RDI produces three forms commonly called the initial value of the Reconnaissance Drought Index (α_k_), the normalized Reconnaissance Drought Index (RDI_n_), and the Standardized Reconnaissance Drought Index (RDI_st_) [57]. RDI is computed using precipitation (P) and potential evapotranspiration (PET), which can be estimated using the Hargreaves-Samani method [17], as expressed in Equation (20):


$$\begin{array}{c} \text{PET}=\text{0}.\text{0023 Ra }\left(\frac{\text{Tmax}+\text{Tmin}}{\text{2}}+\text{17}.\text{8}\right)\sqrt{(\text{Tmax}}-\text{Tmin}) \end{array}$$ (20)

Where: T _max_ and T _min_ represent the maximum and minimum air temperature (^o^C), respectively, and Ra (MJ/m^2^/day) is the extraterrestrial solar radiation, which depends on the latitude of the study area and the time of year.

The initial RDI value (α_k_) is calculated as the ratio of cumulative precipitation to cumulative potential evapotranspiration over a selected timescale k, as shown in Equation (21):


$$\begin{array}{c} {\alpha \text{k}}^{\text{i}}=\frac{\sum_{\text{j}=\text{1}}^{\text{k}}\text{Pij}}{\sum_{\text{j}=\text{1}}^{\text{k}}\text{PETij}},\text{ i}=\text{1},\;\text{2},..,\text{N};\text{ j}=\text{1},\;\text{2},..\text{K} \end{array}$$ (21)

Where: pij and PETij denote precipitation and potential evapotranspiration, respectively, for the j^th^ month of the i^th^ year, N is the total number of years, and αk represents the initial value of the RDI value.

The Normalized values of the Reconnaissance Drought Index (RDIn) is calculated by using equation 22: -


$$\begin{array}{c} \text{RDIn}=\frac{\alpha \text{ki}}{\overset{―}{\alpha_{\text{k}}}}-\text{1} \end{array}$$ (22)

Where: RDIn is normalized Reconnaissance Drought Index, and $\overset{―}{a_{k}}$ is mean of the αk value

The RDI_st_ was initially formulated under the assumption that αk follows a log normal distribution. Accordingly, RDI_st_ is computed using Equation (23)


$$\begin{array}{c} \text{RDIst}=\frac{\text{yi}-\overset{―}{\text{y}}}{\delta \text{y}} \end{array}$$ (23)

Where: y(i)= ln(αk^i^), $\overset{―}{y}$ is the arithmetic mean of y(i), and δy is the standard deviation. The αk values generally follow either the log-normal or gamma distribution at most locations and time scales. However, in many cases, the gamma distribution, provides a better fit to the data. Therefore, the estimation of RDI_st_ is more reliable when the gamma probability density function (pdf) is fitted to the frequency distribution of αk.

Due to a shortage of independent quantitative datasets, a qualitative validation approach was used to test the validity of the suggested exposure risk index in the AC sub-basin, with reference to comparable systems such as the Bilate river watershed. To ensure agreement with known exposure conditions, the index’s spatial patterns were compared with established environmental gradients such as elevation, temperature, drought-prone areas, and proximity to water bodies. High-risk zones were cross-validated against stated drought hotspots in the Wolayta zone and Sidama region, confirming with previous studies. The findings were also interpreted in the context of local environmental information, such as dominating rain-fed agriculture techniques and reported water stress. Furthermore, the index showed its ability to integrate climatic, hydrological, topographic, and socioeconomic characteristics, which providing advantages over existing single-variable indicators. A comparison with established proxies such as rainfall variability, vegetation condition, and soil moisture proved that the index provided better insight into regional exposure risk.

## Results and discussion

### Annual rainfall trend analysis

**Fig 2:**
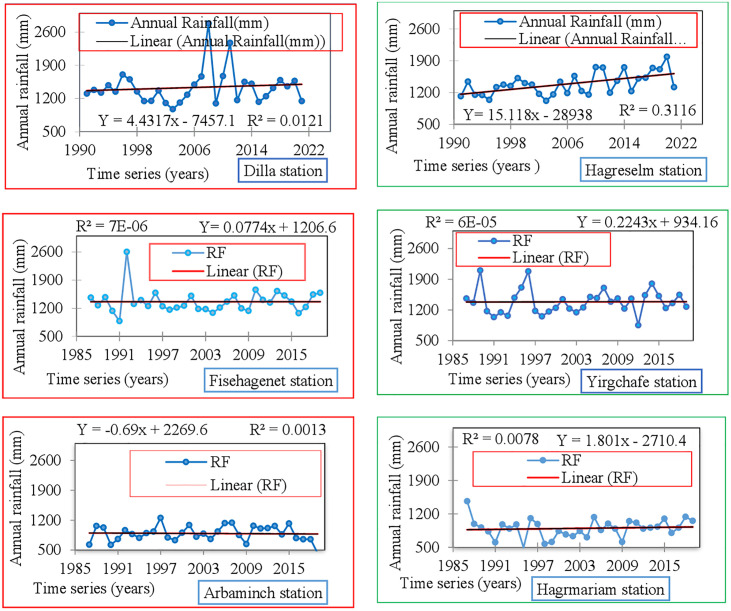
Mean annual rainfall trend of the Abaya-Chamo sub-basin. The figure illustrates long-term variability in mean annual rainfall, with a fitted trend showing temporal changes.

Trend analysis was conducted on annual rainfall and temperature data obtained from six meteorological stations within the watersheds. The Mann-Kendall trend test was applied to the annual precipitation data, and the results for each station during the selected base period are presented in Table 4 and Fig 2. Based on the calculated statistical parameters and chosen level of significance, the null hypothesis was accepted for five stations (Fisehagenet, Hageremariam, Yirgachefe, Arbaminch, and Dilla) and rejected for Hagereselam. Table 4 summarizes the trend statistics for all stations. At five stations (Fisehagenet, Hageremariam, Yirgachefe, Dilla, and Hagereselam), annual rainfall showed an increasing trend, as indicated by positive Mann-Kendall statistics (S) and Sen’s slope values, suggesting a slight rise in rainfall during the selected base period. In contrast, the Arbaminch station exhibited negative values of both the Mann-Kendall statistic and Sen’s slope, suggesting a decreasing trend in annual rainfall. Another calculated parameter, Kendall’s tau, was positive for five stations, indicating a positive correlation, whereas Arbaminch station was negative, suggesting a weak correlation. In addition to the statistical analysis, graphical interpretation was employed to further examine the rainfall trends. The graphical results (Fig 2) show that mean annual rainfall fluctuated slightly across most stations; however, these variations were statistically insignificant for the majority of them.

**Table 4:** Annual rainfall trend analysis for the eastern escarpment of the Abaya Chamo sub basin.

Statistics	Fisehagenet	Hageremariam	Yirgachefe	Arbaminch	Dilla	Hagereselam
Kendall’s tau	0.08	0.148	0.14	−0.008	0.067	0.394
S-value	42	78	74	−4	31	183
P-value	0.53	0.235	0.261	0.96	0.613	0.002
Var (S)	0	0	0	0	0	0
Alpha (α)	0.05	0.05	0.05	0.05	0.05	0.05
Sen’s slope	2.17	3.74	4.62	−0.16	3	15.02

For Fisehagenet, the mean annual rainfall trend showed that the coefficient of determination (R^2^) was 0.0007%, indicating almost no fit along the line of best fit. Similarly, graphical analysis of Hageremariam, Yirgachefe, Dilla, and Hagereselam showed rainfall trends, with R^2^ values of 0.78%, 0.006%, 1.21%, and 31.6%, respectively. At Arbaminch station, the graph shows that annual rainfall decreased slightly between 1987 and 2019, with R^2^ of 0.13%, indicating a very weak fit along the line of best fit. Overall, the annual mean precipitation graphs indicate that Dilla and Hagereselam exhibited relatively higher increasing trends in annual rainfall compared to the other stations (Fig 2). These results suggest that, although rainfall trends were generally weak and statistically insignificant across most stations, Dilla and Hagereselam experienced a comparatively more noticeable increase in annual precipitation during the study period.

Numerous studies have analyzed rainfall trends across various watersheds and reported slight increases or decreases in annual rainfall over their respective study periods. For example, a time-series analysis of rainfall trends in the Woleka sub-basin of north-central Ethiopia revealed a declining trend in annual rainfall [30]. A similar study conducted in the Rift Valley Lake Basin found no statistically significant trends in rainfall across the region [71]. In contrast, analyses of seasonal and annual rainfall and temperature data over southern Ethiopia revealed substantial variability, contributing to recurrent droughts and floods [72]. Likewise, a study conducted at the Illala meteorological station, utilizing the Mann-Kendall test and Sen’s slope estimator from 1995–2014 time series, identified a slight decreases in precipitation and highly variable annual and seasonal temperature patterns, even at localized spatial and temporal scales [73].

### Mean monthly maximum and minimum temperature trend analysis

#### Mean monthly maximum temperature.

Using the Mann-Kendall trend test on the mean monthly maximum temperature, the null hypothesis was rejected for five stations (Fisehagenet, Hageremariam, Arbaminch, Dilla, and Hagereselam) and accepted for Yirgachefe. Sen’s slope estimates were positive for all stations except at Dilla, which exhibited a negative slope, indicating a slight decline in mean monthly maximum temperature at that station. Despite the negative Sen’s slope observed at Dilla, the Mann-Kendall test statistics (S) indicated increasing trends in maximum temperature at most stations, suggesting an overall upward trend in mean monthly maximum temperature during the study period. Furthermore, Kendall’s tau values were positive for the five stations, implying a strong positive correlation between temperature and time, whereas Dilla station showed a negative tau value, indicating a weak or negative correlation (Table 5).

**Table 5:** Result of the Mann-Kendall trend test for mean monthly maximum temperature.

Statistics	Fisehagenet	Hageremariam	Yirgachefe	Arbaminch	Dilla	Hagereselam
Kendall’s tau	0.61	0.436	0.197	0.477	−0.265	0.572
S-value	322	230	140	236	123	249
P-value	<0.0001	0	0.111	0	0.038	<0.0001
Var (S)	0	0	0	0	0	0
Alpha (α)	0.05	0.05	0.05	0.05	0.05	0.05
Sen’s slope	0.102	0.033	0.023	0.04	−0.021	0.112

**Fig 3:**
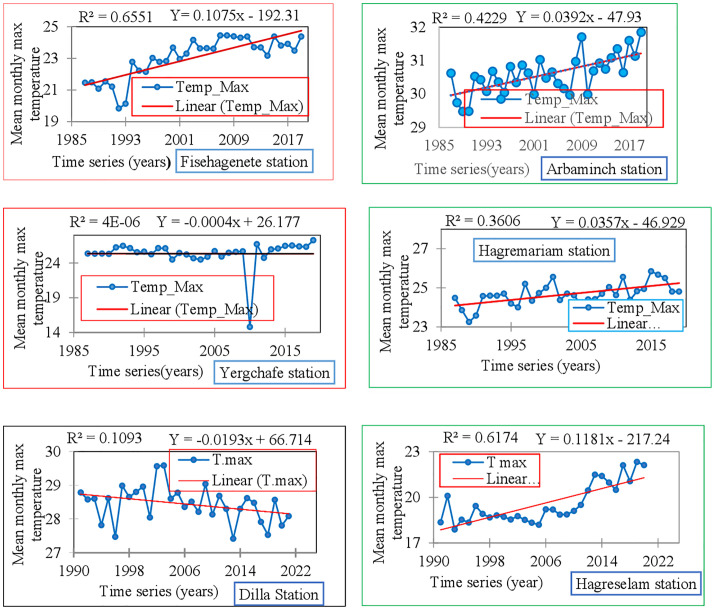
Mean monthly maximum temperature trend in the eastern escarpment of the Abaya Chamo sub-basin. The figure illustrates temporal variability in mean monthly maximum temperature.

To provide further insight, graphical interpretation, linear regression analysis, and slope estimation were performed, and the results are presented for each station (Fig 3). For example, the graphical analysis of the mean monthly maximum temperature at Fisehagenet showed an increasing trend, with the coefficient of determination (R^2^) indicating that 65.51% of the variability in temperature was explained by the line of best fit. Similarly, the graphs for Hageremariam, Arbaminch, and Hagereselam also showed increasing trends, with R^2^ values of 36.06%, 42.29%, and 61.74%, respectively. In contrast, Dilla and Yirgachefe exhibited slight decreasing trends in mean monthly maximum temperature, with R^2^ values of 10.93% and 0.0004%, respectively, indicating very weak linear relationships. Overall, the combined results of the Mann-Kendall trend test and Sen’s slope estimator indicated that mean monthly maximum temperatures in the basin increased at five stations and slightly decreased at one station over the selected base period (Table 5).

#### Mean monthly minimum temperature.

The Mann-Kendall trend test was applied to the mean monthly minimum temperature data from six stations, and the results are presented in Table 6. The null hypothesis was accepted for three stations (Arbaminch, Hageremariam, and Dilla) and rejected for the remaining three stations (Fisehagenet, Yirgachefe, and Hagereselam). Overall, the analysis indicated an increase in mean monthly minimum temperature across the study area during the observation period. However, a slight decrease was observed at Hageremariam and Dilla, as evidenced by negative S and Sen’s slope values. Kendall’s tau was negative for three stations (Hageremariam, Hagereselam, and Dilla), indicating weak or negative correlations with time, whereas it was positive for the other three stations (Arbaminch, Fisehagenet, and Yirgachefe), indicating stronger positive correlations (Table 6).

**Table 6:** Result of the Mann-Kendall trend test for mean monthly minimum temperature.

Statistics	Fisehagenet	Hageremariam	Yirgachefe	Arbaminch	Dilla	Hagereselam
Kendall’s tau	0.583	−0.197	0.419	0.02	−0.161	−0.406
S-value	308	−104	221	1	75	189
P-value	<0.001	0.111	0.001	1	0.211	0.001
Var (S)	0	0	4164.3	4164.3	0	0
Alpha (α)	0.05	0.05	0.05	0.05	0.05	0.05
Sen’s slope	0.058	−0.041	0.039	0.000063	−0.148	−0.148

**Fig 4:**
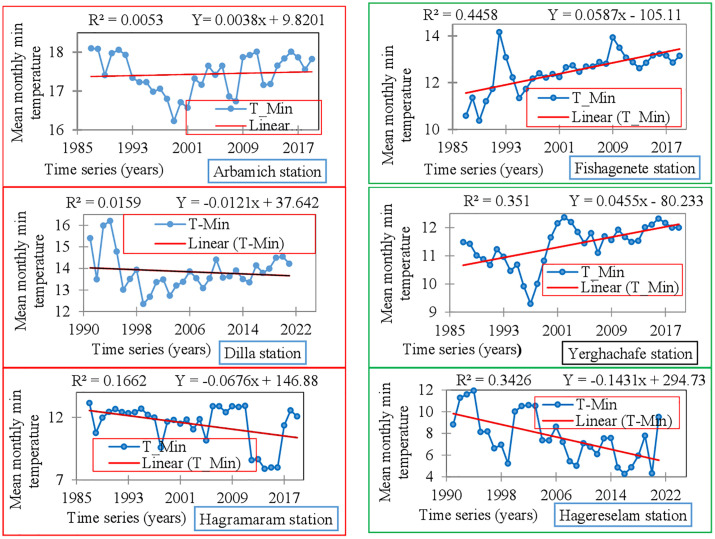
Mean monthly minimum temperature trend in the eastern escarpment of the Abaya-Chamo sub-basin. The figure illustrates temporal variability in men monthly minimum temperature.

The graphical analysis of the basin revealed variability in the R^2^ across different stations. At Fisehagenet, approximately 44.58% of the variation in mean monthly minimum temperature was explained by the fitted trend line. Similarly, the Hageremariam, Dilla, and Hagereselam showed slight month-to-month fluctuations in temperature, with R^2^ values of 16.62%, 1.59%, and 34.26%, respectively. In contrast, Arbaminch and Yirgachefe exhibited increasing trends, with R^2^ values of 1.59% and 35.1%, respectively, indicating varying degrees of linear fit (Fig 4). Overall, the combined results of the Mann-Kendall trend test and Sen’s slope estimator indicated that mean monthly minimum temperatures across the basin experienced both slight increases and decreases over the selected base period.

Comparable studies conducted in several basins worldwide have documented varying degrees of temperature variability. The findings of the present study are consistent with those of studies conducted in the Rift Valley Lake Basin, which reported increases in both maximum and minimum temperatures at varying rates [28,74,75]. Similarly, a study examining temperature and precipitation extremes in the Wolaita Zone of southern Ethiopia found positive trends in annual maximum and minimum temperatures [76]. Moreover, studies on daily temperature and precipitation extremes conducted across different agroecological zones in the Gurage Zone of south-central Ethiopia reported statistically significant increases in the number of warm days and nights, alongside significant decreases in the number of cold days and nights. These findings indicate a persistent warming trend across multiple watersheds in the region [77].

Similar results have been reported in other parts of Ethiopia, where mean annual temperatures showed an increasing trend during the period from 1984 to 2006 [10]. Another study also reported that, due to increasing human pressure, extreme weather events and climatic change have contributed to a continuous rise in temperature [75]. Research conducted in Ethiopia’s Central Rift Valley Basin also identified increasing temperature trends ranging from 0.12 to 0.54 °C per decade across different locations [78]. In the southeastern part of the sub-basin, a study examining the spatiotemporal variability of hydroclimatic variables found that annual minimum temperature increased by 0.05 to 0.5 °C in some areas, while decreases of 0.1 to 0.3 °C were observed in others [47]. Furthermore, the National Adaptation Programme of Action (NAPA) for Ethiopia reported that temperatures across the country have risen in accordance with broader global and African warming trends [79].

### Streamflow data

Using the statistical parameters of the median and coefficient of dispersion (CD), streamflow variability was assessed using Indicators of Hydrologic Alteration (IHA) method, and the results are presented in Table 7. At the gauging station, significant hydrologic alterations were observed in IHA Group 1, where mean streamflow increased significantly from July to November, while slight increases or decreases were observed during the remaining months. Higher median flows were observed during May, June, October and November, reflecting the influence of the main rainy season, while dry months have lower median flows, especially in February and March.

**Table 7:** IHA results for the Eastern escarpment of Abaya Chamo sub-basin at Tore gauging station.

IHA Group 1	Unit	µ	CD	IHA Group 2	µ	CD	IHA Group 3	µ	CD
January	m^3^/s	1.514	0.573	1-day minimum	0.492	0.674	Date of minimum	88.5	0.103
February	m^3^/s	0.981	0.684	3-day minimum	0.583	0.682	Date of Maximum	280.5	0.41
March	m^3^/s	0.821	1.178	7-day minimum	0.611	0.628	IHA Group 4	µ	CD
April	m^3^/s	1.844	1.704	30-day minimum	0.736	0.457	Low pulse count	2.5	1.2
May	m^3^/s	8.864	0.642	90-day minimum	1.145	0.715	Low pulse duration	13	2.89
June	m^3^/s	6.014	0.848	1-day maximum	15.84	0.267	High pulse count	5	0.55
July	m^3^/s	3.188	0.788	3-day maximum	15.7	0.266	High pulse duration	7.25	1.259
August	m^3^/s	2.77	1.024	7-day maximum	15.09	0.256	IHA Group 5	µ	CD
September	m^3^/s	3.726	1.166	30-day maximum	12.58	0.293	Rise rate	0.25	0.48
October	m^3^/s	10.28	0.687	90-day minimum	9.034	0.330	Fall rate	−0.15	−0.39
November	m^3^/s	5.697	1.119				Numbers of reversals	70	0.28
December	m^3^/s	2.566	0.784						

Where: (µ=median, CD=Coefficient of dispersion).

The coefficient of dispersion is relatively high in several months (April, September, and November), showing significant interannual variability in monthly flows and sensitivity to climate variability and watershed responses. For IHA Group 2, the duration of annual minimum flows increased, while the duration of maximum flow decreased. The minimum flow events (1-day to 90-day minimum) show comparatively low median values with notable dispersion, implying variability in low flow persistence, as indicated in Table 7. In contrast, the high median value (exceeding 15 m^3^/s) and relatively low CD values of the maximum flow indicators (1-day, 3-day, and 7-day maxima) suggested that peak flow events were more stable in magnitude across years, possibly due to consistent seasonal rainfall patterns.

IHA Group 3 (timing of annual extreme flows) showed fewer significant hydrologic alterations. The median timing of minimum flow occurred around day 88.5 (late March), while the median maximum flow occurred around day 280.5 (early October). This timing was consistent with the basin’s seasonal rainfall pattern, in which low flows dominated during the dry season and peak flows occurred during the main rainy season. The moderate dispersion in the timing of flows indicated some interannual variability in the onset and intensity of peak rainfall.

IHA Group 4 (frequency and duration of high and low pulses) illustrated that low-flow pulses occurred frequently and persisted for longer durations (median duration of 13 days), whereas high-flow pulses occurred more frequently (median count of 5) but have shorter durations (7.25 days). The high CD values for pulse duration suggest that both high- and low-flow conditions were highly variable, which may have influenced aquatic habitats and sediment transport processes. IHA Group 5 (rate and frequency of flow variations) showed comparatively low rise and fall rates, indicating gradual variations in streamflow rather than abrupt fluctuations. The relatively high number of flow reversals (70) suggested frequent transitions between rising and falling limbs of the hydrograph, which may have been be attributed to rainfall variability, land-use changes, or watershed management influences.

Overall, the study identified a decrease in high flows and small floods, accompanied by an increase in extreme low flows. Using the IHA method, the results in Table 7 were classified into five key aspects of hydrologic alteration: monthly streamflow magnitude, magnitude and duration of annual extremes, timing of annual extremes, frequency and duration of high and low pulses, and rate and frequency of flow changes. The findings from all five groups revealed significant variability in streamflow over time, highlighting dynamic changes in the watershed’s hydrologic regime.

A general trend analysis of river streamflow at Tore gauging station revealed a clear declining trend, providing valuable insights for policymakers developing climate change adaptation and mitigation strategies for local farmers. This decline was particularly important for managing climate variability, as many of the observed changes were driven by anthropogenic factors, including agricultural practices, land-use change, urbanization, deforestation for charcoal production, and expansion of settlements. Results from the individual IHA groups indicated a significant increase in the frequency of low flow pulses and flow reversals, suggesting that such events become more frequent over time. Furthermore, the coefficient of dispersion, a non-parametric measure of interquartile spread normalized by the median, was calculated for each IHA group using reference period values. The results, presented in Table 7, demonstrated the percent change from the reference period, underscoring the extent of IHA within the watershed.

Similar findings have been reported in numerous studies across different watersheds, demonstrating substantial variability in streamflow under diverse environmental and anthropogenic conditions [80]. Investigating the variability of rainfall, temperature, and river discharge using long-term time-series data has provided critical insights into the occurrence of extreme hydrological events such as droughts and floods. Such analyses have been essential for effective water resource management, particularly in regions highly exposed to climate variability, as they have supported support the sustainable development and management of watersheds [81]. For example, a study assessing streamflow trends across the Upper Blue Nile basin using data from three stations along the main river stem between 1964 and 2003, reported a significant increase in annual streamflow during the rainy season, accompanied by a decline during the dry season [82].

Similarly, studies conducted in the Awash River Basin revealed a notable decreasing trend in both annual and seasonal streamflow at the Modjo gauging station. These findings highlighted the importance of continuous monitoring and analysis of streamflow and climatic variables to inform adaptive water resource management strategies [83]. Early identification of shifting hydrological patterns enabled stakeholders to implement proactive measures such as optimized reservoir operations, early warning systems, and drought and flood preparedness plans. These measures enhanced resilience to climate-induced extremes and supported the long-term sustainability of vulnerable river basins.

### Standardized precipitation index (SPI) and Reconnaissance drought index (RDI)

**Fig 5:**
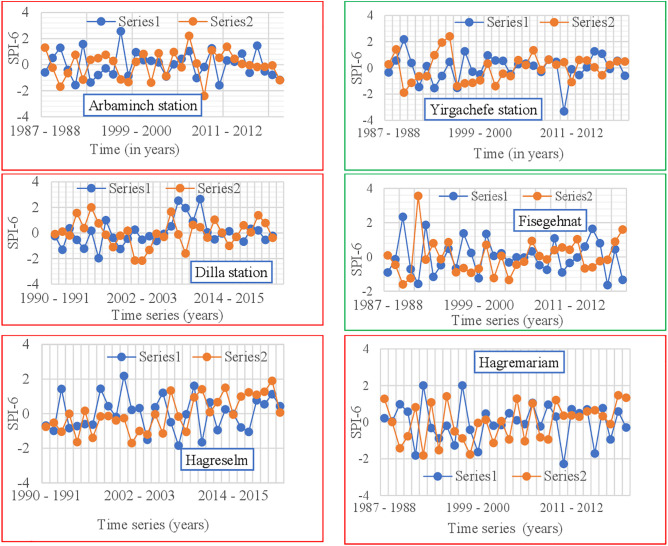
Six-month Standardized Precipitation Index (SPI-6) for the Easter escarpment of the Abaya-Chamo sub-basin. Series 1: October-March; series 2: April-September: Drought patterns are shown for each series.

**Fig 6:**
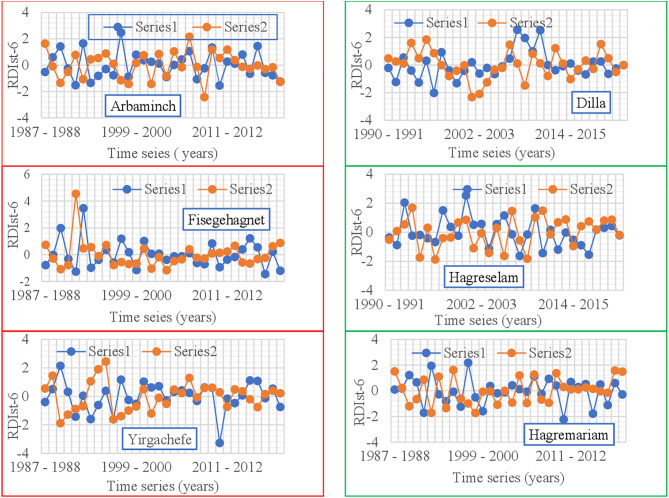
Six-month Reconnaissance Drought Index (RDI_st_-6) for the Eastern escarpment of the Abaya-Chamo sub-basin. Series 1: October-March; Series 2: April-September: Drought patterns are shown for each series.

The Standardized Precipitation Index (SPI) and Reconnaissance Drought Index (RDI) were used to assess long-term drought trends and examine the historical occurrence of droughts over extended time scales. In this study, the DrinC software package was employed to calculate the regional SPI and RDI characteristics using six- and twelve-month climate data time series. Each district exhibited variations in temperature and rainfall across different years, resulting in distinct and severe drought events during specific periods. The results derived from SPI-6, SPI-12, RDI-6, and RDI-12 are presented in Figs 5 and 6, highlighting variations in drought severity and frequency across the study area.

#### Drought analysis for SPI-6.

The SPI-6 index, calculated for the first six-month rolling period (October**-**March) using 33 years of data from the Arbaminch station, revealed significant drought patterns. Specifically, it identified three moderate drought years (1993–1994, 2007–2008, and 2018–2019) and two severe drought years (1995–1996 and 2010–2011), as shown in Fig 5. Similarly, the SPI-6 calculated for the second rolling period (April-September) identified six moderate dry years (1987–1988, 1992–1993, 1997–1998, 1998−1999, 2001−2002, and 2018−2019), one severe drought year (1989**−**1990), and one extreme drought year (2008–2009), with most years remaining within near normal. Notably, the years 2008–2009 and 2010–2011 recorded the most severe drought conditions, with SPI values of −1.69 and −2.41, respectively, indicating consecutive years of pronounced drought severity. These findings underscore the variability and intensity of drought events in the region, with certain years exhibiting extreme and persistent dry conditions.

At the Fisehagenet station, the first series of SPI-6 rolling period revealed four moderate drought years (1993–1994, 1997–1998, 1999–2000, and 2018–2019), and two severe drought years (1991–1992 and 2016–2017), with no extreme drought events observed. For the second rolling series, three moderate drought years (1990–1991, 2001–2002, and 2003–2004) and one severe drought year (1989–1990), were identified, again with no extreme drought events. The most intense droughts occurred in 2016–2017 and 2018–2019, with SPI values of −1.65 and −1.60, respectively (Fig 5). At the Yirgachefe station, the first SPI-6 rolling period indicated moderate drought conditions in 1991–1992, a severe drought in 1993–1994, and an extreme drought event in 2010–2011. The second rolling period revealed five moderate drought years (1990–1991, 1996–1997, 1997–1998, 1999–2000, and 2001–2002) and one severe drought year (1989–1990), with no extreme drought events.

The most severe droughts occurred in 1989−1990 and 2010−2011, with SPI values of **−**1.89 and **−**3.31, respectively (Fig 5). These findings highlight the variability and severity of drought conditions across different years. At the Hageremariam station, SPI-6 values calculated over 33 years for the first rolling series revealed three severe drought years (1991−1992,1999−2000, and 2014−2015), one extreme drought year (2010−2011), and one moderate drought year (1996−1997). For the second rolling period, three moderate drought years (1989**−**1990, 2001**−**2002, and 2005**−**2006) and three severe drought years (1992**−**1993, 1994**−**1995, and 1998−1999) were identified. The highest drought indices were **−**1.81 and **−**2.27, respectively, for 1992–1993 and 2010–2011.

For the Dilla station, SPI-6 values calculated over 32 years (1990−2021) for the first rolling series showed no moderate drought events but identified two severe drought years (1996**−**1997 and 2008**−**2009), with no extreme drought events observed. Similarly, during the second SPI-6 rolling period, three moderate drought years (1998−1999, 2003**−**2004, and 2014**−**2015), two severe drought years (1993−1994 and 2008−2009), and two extreme drought years (2001**−**2003) were identified. The most intense droughts occurred in 1996**−**1997 and 2002**−**2003, with SPI values of **−**1.97 and **−**2.17, respectively, representing the highest drought severity recorded indices across the two rolling periods.

At the Hagereselam station, SPI-6 analysis over 32 years revealed four moderate drought years (2005–2006, 2007–2008, 2010–2011, and 2016–2017) and one severe drought year (2003−2004) in the first rolling series, with no extreme drought events and predominantly near-normal conditions. In the second rolling series, eight moderate drought years were identified (1992−1993,1994−1995,1996−1997,2002−2004,2005−2006, and 2008−2009). The most severe droughts occurred in 2007−2008 and 2001−2002, with SPI values of −1.85 and −1.7, respectively.

Overall, the SPI-6 analysis across multiple stations in the Eastern escarpment of the AC sub-basin over the 32–33-year period revealed pronounced spatial and temporal variability in drought frequency and severity. Moderate to extreme drought events were particularly prominent during the early 1990s, late 2000s, and early 2010s, with noticeable seasonal differences between the October**-**March and April**-**September periods. The repeated occurrence of droughts of varying intensity across stations highlighted the region’s vulnerability to prolonged dry spells, while the identification of the timing and spatial distribution of the most severe drought events provided valuable insights for targeted seasonal planning and regional water resources management.

#### Drought analysis for RDI_st-_6.

The RDI_st_-6 calculated for the first six rolling month series at Arbaminch station, four moderate dry conditions (1993−1994, 2007−2008, 2009−2010, and 2018−2019) and two severe drought years (1991−1992 and 2010−2011) were identified, with no extreme drought years observed. For the second six-month rolling month series, seven moderate drought years (1989−1990, 1992−1993, 1997−1999, 2001−2002, 2009−2010, and 2018−2019) and one extreme drought year (2008−2009) were observed, as shown in Fig 6. The most severe droughts occurred in 2010−2011 and 2008−2009, with RDI values of −1.55 and −2.44, respectively. The RDI_st_-6 calculated for the first six rolling months series at Fisehagenet station showed four moderate drought years (1991−1992, 1999−2000, 2010−2011, and 2018−2019) and no severe or extreme drought events. For the second rolling period, three moderate drought years (1989−1990, 2001−2002, and 2007−2008) were identified, again with no severe or extreme drought events were identified. The highest drought indices occurred in 2016−2017 and 2003−2004, with RDI values of −1.45 and −1.17, respectively.

The first six-month rolling series of the RDI_st_-6 for Hageremariam station revealed two instances of moderate drought conditions (1996−1997 and 2015−2016), one extreme dry event (2010−2011), and no severe dry conditions. In the second rolling series, three extreme dry events were observed (1989−1990, 1994−1995, and 1987−1988), along with one severe dry year (1992−1993), but no extreme dry conditions. The highest drought indices for these periods were −2.22 for 2010−2011 and −1.73 for 1998–1999, marking the most severe drought conditions in the respective years. For Yirgachefe station, the first six-month rolling series of RDI_st_-6 revealed one moderate dry event (1991−1992), two severe dry conditions (1993−1994 and 1996−1997), and one extreme dry event (2010−2011). In the second rolling series, two severe drought years (1989−1990 and 1996−1997) and five moderate dry years (1989−1990, 1992−1993, 1997−1998, 1998−1999, 2001−2002, and 2018−2019) were identified, with no extreme dry events recorded. The most extreme drought indices were −3.27 for 2010–2011 and −1.88 for 1989–1990, as shown in Fig 6. These findings highlight the varying severity and frequency of drought events across different stations, with the most extreme drought conditions observed in 2010–2011 and 1989–1990.

For the Dilla station, the RDI_st_-6 values calculated over 32 years for the first six-months rolling revealed three moderate drought years (1991−1992, 1994−1995, 1999−2000) and one extreme drought years (1996−1997). In the second rolling series, three moderate drought years (2003−2004, 2008−2009, and 2014−2015) and one extreme drought years (2001–2003) were observed. The most severe droughts occurred in 2001–2003, with RDI values of **−**2.1 and **−**2.33, respectively (Fig 6). At the Hagereselam station, the RDI_st_-6 for the first six- month rolling period showed three moderate drought years (2003–2004, 2010–2011, and 2012–2013), two severe drought years (2007–2008, and 2016–2017), and no extreme dry events. In the second rolling series, two moderate drought years (2001–2002, and 2003–2004) and four severe dry conditions (1994–1995, 1996–1997, 2005–2006, and 2008–2009) were identified, with no extreme drought events. The highest drought indices were recorded in 2007**−**2008 and 1996−1997, with RDI values of −1.65 and −1.89, respectively, across both series (Fig 6).

These results highlighted the spatial and temporal variability in drought intensity and frequency across stations, with some years experiencing extreme and severe drought events. Overall, the 32-to-33-year RDI_st_-6 analysis of stations in the Abaya-Chamo sub-basin illustrated substantial regional and seasonal variability in drought intensity. Significant drought events occurred in 2010–2011, 2008–2009, and 1989–1990, with moderate to severe conditions observed across most stations. These findings emphasized the region’s susceptibility to prolonged dry periods and provided valuable insights for targeted drought management and climate resilience planning.

#### Drought analysis for SPI and RD_Ist_-12.

The SPI-12 rolling analysis for the Arbaminch station, calculated over a 12-month period (October–September), revealed one extreme drought year (1998−1999), one moderate dry year (2003−2004), and one severe drought year (2008−2009). At the Fisehagenet station, the SPI-12 analysis showed four moderate drought years (1996−1997, 1999−2000, 2001−2002, and 2016−2017) and two severe drought years (1990−1991, 2003−2004), with no extreme events observed. At the Yirgachefe station, the SPI-12 results revealed five moderate drought years (1994−1995, 1998−1999, 1999−2000, 2010−2011, and 2011−2012), two severe drought years (1988−1989, 1991−1992), and one extreme drought year (1996−1997). The maximum drought index for Fisehagenet was −1.68 in 1990–1991, while Yirgachefe recorded the highest index of −2.31 in 1996–1997.

**Fig 7:**
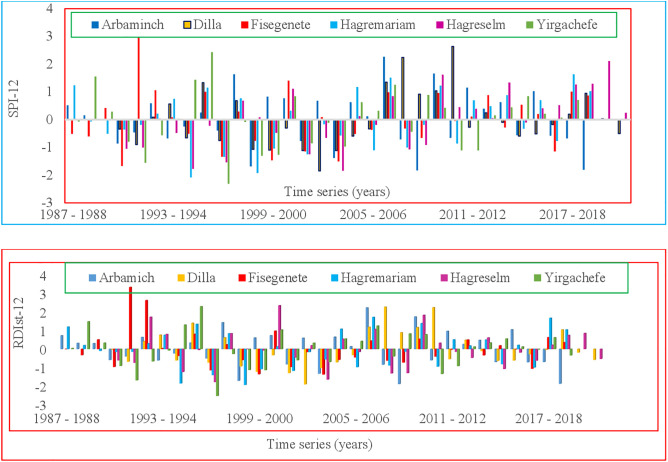
Comparison of 12-month SPI and RDI_st_ depicting drought conditions in the Eastern escarpment of the Abaya-Chamo sub-basin. The figure highlights the similarities and differences between the two indices in capturing drought severity and temporal variability.

From 1987 to 2019, the SPI-12 analysis at the Hageremariam station indicated six moderate drought years (1996−1997, 1999−2000, 2001−2002, 2005−2006, 2000−2001, 2007−2008), no severe dry conditions, and one extreme drought year (1994−1995). At the Dilla station, the SPI-12 rolling series revealed five moderate drought years (1998−1999, 1999−2000, 2001−2002, 2003−2004, 2009−2010), and one severe drought year (2002−2003), with no extreme drought events observed. The SPI-12 results for Hagereselam indicated four moderate dry years (1990–1992, 2001–2002, 2007–2008) and three severe drought periods (1994−1996 and 2003–2004), as shown in Fig 7. The most extreme drought indices were recorded in 1994−1995 at Hageremariam (−2.08) and in 2003−2004 at both Hagereselam and Dilla stations (−1.84). These findings underscore the spatial variability in drought intensity and frequency across the stations, with certain periods experiencing severe to extreme sever.

The RDI_st_-12 analysis for the Arbaminch station indicated one moderate drought year (2003–2004) and three severe drought years (1998–1999, 2007–2008, and 2017–2018). In contrast, the RDI_st_-12 results for the Fisehagenet station revealed four moderate drought years (1996–1997, 1999–2000, 2003–2004, and 2016–2017), with no severe or extreme drought events recorded. At the Yirgachefe station, the RDI_st_-12 analysis showed three moderate droughts (1998–2000 and 2010–2011), one severe drought year (1996–1997), and one extreme drought event (1991–1992). For the Hageremariam station, the RDI_st_ result indicated one severe drought year (1998–1999), while moderate drought conditions occurred in 1996–1997,1999–2000, and 2002–2003. At the Dilla station, three moderate drought years (1999–2000, 2001–2002, and 2003–2004), one severe drought year (2002–2003), and one extreme drought year (2007–2008) were identified over the 32-year period from 1990 to 2021. Similarly, the 32-year RDI_st_-12 rolling series for the Hagereselam station showed four moderate drought conditions (1994–1995, 2007–2009, and 2015–2016) and one severe drought year (2003–2004), with no extreme drought events observed. The most severe RDI_st_-12 drought index values were recorded as −1.88 at Arbaminch (2007–2008), −1.37 at Fisehagenet (2003–2004), −2.52 at Yirgachefe (2003–2004), −2.52 at Yirgachefe (1996–1997), −1.77 at Hageremariam (1996–1997), −1.93 at Dilla (2002–2003), and −1.77 at Hagereselam (2002–2003).

As evidenced by the results across all stations, drought occurrence was not continuous over time at any single station. The SPI-6 analysis indicated that drought events occurred at all stations, although the magnitude and frequency varied between the two rolling periods. In most cases, the second rolling period exhibited a higher frequency of drought events than the first. Arbaminch and Hageremariam stations experienced the highest frequency of drought events among all stations. Notably, the years 2010−2011 at Arbaminch, Hageremariam, and Yirgachefe, and 2002−2003 at Dilla recorded the most severe drought indices, with SPI-6 values of −2.41, −2.27, −3.31, and −2.17, respectively. Similarly, the RDI_st_-6 analysis revealed that all stations experienced drought events during different drought years throughout the study period, with the highest frequency of drought conditions observed at the Arbaminch and Hagereselam stations.

The most extreme RDI_st_-6 drought indices were recorded at Yirgachefe (−2.44) in 2008−2009 and at Arbaminch (−3.27) in 2010−2011. Overall, the six-month drought analysis using SPI-6 and RDI_st_-6 across multiple stations in the eastern escarpment of the Abaya-Chamo sub-basin demonstrated pronounced spatial and temporal variability in drought frequency and severity throughout the study period. Both indices consistently indicated significant drought events, with 2010−2011, 2008−2009, and 1989−1990 emerging as severe to extreme drought periods. The SPI-6 generally indicated more frequent and intense extreme droughts, such as the extreme drought observed at Yirgachefe in 2010–2011 (−3.31), reflecting its high sensitivity to precipitation deficits.

Conversely, RDI_st_-6 index showed fewer extreme drought events but highlighted a broader distribution of moderate to severe droughts, reflecting the integration of rainfall deficit measures that may moderate the classification of extremes. Seasonal patterns were also evident, with the October-March periods exhibiting sharper peaks in drought severity, whereas the April-September periods showed more frequent occurrence of moderate drought across stations such as Arbaminch, Hageremariam, and Dilla. The concurrence of severe drought years identified by both indices reinforces the robustness of the findings, and highlights the region’s vulnerability to prolonged dry spells. These results underscore the need for targeted drought mitigation and improved water resource management strategies.

These results underscore the importance of using complementary drought indices to capture the complexity of drought dynamics and inform effective climate resilience planning in the Abaya-Chamo sub-basin. The SPI-12 analysis across all stations revealed that moderate drought conditions occurred most frequently at each station. However, Hageremariam and Yirgachefe recorded the most extreme drought indices, with values of −2.31 in 1996–1997 and −2.08 in 1994–1995, respectively. Similarly, the RDI_st_-12 analysis indicated the most severe conditions at Yirgachefe in 1996–1997 (−2.52) and at Dilla in 2002−2003 (−1.93).

**Fig 8:**
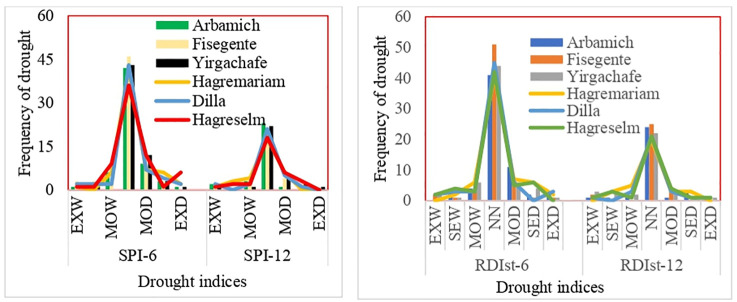
Frequency distribution of drought indices for Eastern escarpment of Abaya-Chamo sub-basin. The figure shows the distribution of drought index values and their frequency of occurrence**.**

Both SPI-12 and RDI_st_-12 analyses revealed substantial spatial variability in drought characteristics among the stations. The SPI-12 index identified more frequent and intense moderate to extreme drought events, whereas the RDI_st_-12 index detected fewer extreme events but a greater occurrence of moderate and severe droughts. Overall, SPI-12 demonstrated increased drought frequency and severity, particularly at stations such as Arbaminch and Hageremariam, reinforcing its usefulness for comprehensive drought assessment. Furthermore, the analysis showed that the six-month time scale (SPI-6 and RDI_st_-6) captured a higher frequency of drought occurrences than the twelve-month time scales (SPI-12 and RDI_st_-12), as illustrated in Fig 8. This highlights the greater sensitivity of shorter time scales in detecting drought conditions.

The usefulness of the identified exposure risk index in describing actual exposure patterns across the Abaya–Chamo sub-basin and similar Ethiopian basins is confirmed by the indirect validation. With good spatial consistency, high index values primarily appeared in lowland, high-temperatures zones, and drought-prone areas near Lake Abaya and Lake Chamo, reflecting persistent climatic stress and limited water availability. Reduced water availability and increased evapotranspiration can limit agricultural productivity and reliable access to water for domestic and livestock use in these densely populated zones [71,84–87]. Local knowledge contributes to these findings, with high-index communities frequently experiencing water shortages and crop failures, affecting irrigation and water availability for household and livestock use, while higher-elevation and vegetated areas show lower exposure levels, indicating consistency with reported socio-environmental conditions. This is consistent agrees with studies from similar Ethiopian basins linking drought exposure to elevation, land use, and rainfall variability. The exposure risk index corresponds with established vulnerability regions in the Wolayta Zone and Sidama Region, which have been identified as ecologically sensitive and drought-prone [88,89].

Compared with conventional single-variable indicators, the index integrates climatic, hydrological, topographic, and socio-economic factors, enabling identification of compound high-risk zones and transitional areas that are not captured by rainfall or vegetation indices alone [90,91]. Additionally, the index detects emerging risk areas driven by land-use change and population pressure, supporting early detection of increasing exposure [87,92]. These findings highlight the need for targeted mitigation measures, including improved irrigation efficiency, rainwater harvesting, watershed management, and enhanced rural water supply systems [93,94].

The exposure risk index also provides actionable guidance for planning in the Abaya-Chamo sub-basin. High-risk zones can be prioritized for irrigation improvements, rainwater harvesting, and rural water supply, while emerging risk areas can inform early warning and land-use planning. By integrating climatic, hydrological, topographic, and socio-economic factors, the index offers more precise, multi-dimensional insight than single-variable indicators, supporting evidence-based decision-making for drought preparedness and climate-resilient development. Overall, the validation confirms that the index reliably reflects environmental and socio-economic vulnerabilities, aligns with existing literature and local observations, and provides added insight beyond traditional metrics, making it a reliable tool for exposure assessment in the Abaya-Chamo sub-basin and similar Ethiopian watersheds.

Significant hydroclimatic changes have been observed in southern Ethiopia, characterized by rising temperatures, altered streamflow patterns, and increasing drought severity. These changes were consistent with the influences of large-scale climate drivers such as ENSO (El Niño–Southern Oscillation), NAO (North Atlantic Oscillation), AMO (Atlantic Multidecadal Oscillation), and IOD (Indian Ocean Dipole), which modulate rainfall and temperature variability in the region [95–97]. ENSO phases can either decrease or increase rainfall by altering moisture transport and seasonal rainfall patterns, whereas positive or negative IOD events often exert a stronger influence on regional precipitation variability. Furthermore, Volcanic aerosols may temporarily cool the Earth’s surface and disrupt atmospheric circulation, potentially inducing El Niño-like conditions that reduce monsoon rainfall and increase the frequency and intensity of drought events [95,97,98]. Volcanic eruptions also influence ENSO dynamics, often causing triggering El Niño like conditions that reduce monsoon rainfall and exacerbate drought severity [95,98,99]. The observed hydroclimatic alteration is likely the result of both natural climatic variability and anthropogenic forcing, underlining the need for integrated climate and water resource management approaches in southern Ethiopia [60–63,97,100].

Previous studies indicate that severe dry conditions have become more frequent in recent decades, reflecting increasing hydroclimatic variability and a shift toward higher water deficit relative to the long-term climatic norm [101,102]. The drought sensitivity results based on RDI in this study showed more pronounced variability compared to the SPI, which is consistent with findings from other catchment-based studies. RDI is considered more sensitive than SPI because it incorporates temperature (via potential evapotranspiration) in addition to precipitation, whereas SPI relies solely on precipitation data [103,104]. Similar studies in northeastern Algeria summarize report that, although SPI and RDI exhibit coherent and comparable behavior, RDI shows smaller differences across climate zones and time scales, which is considered over SPI and is attributed to the inclusion of PET in RDI calculations [105].

Likewise, a study in northwestern Morocco for the period 1971–1972–2010–2011 found that drought frequency varies with time scale (3,6, and 12 months); however, correlation analysis revealed a strong positive relationship between SPI and SDI at the 12 month time scale across different periods [106]. In the present study, the proportion of wet events identified by RDI_st_ is higher than that detected by SPI at most time scales, which aligns with previous findings. In contrast, SPI identifies a higher proportion of moderate, severe, and extreme drought events compared to wetness events [48,105]. Analysis of the graphical results indicates that the 12-month indices provide more reliable and representative drought characterization than shorter time scales, as illustrated in Fig 6. Therefore, the use of 12-month SPI and RDI indices is recommended for drought assessment and for the formation of effective mitigation and adaptation measures. Furthermore, global sea surface temperature variability, along with seasonal and rainfall characteristics, may have contributed to the observed differences in drought frequency and temporal scale. Drought severity and hydroclimatic variability in the Abaya -Chamo sub-basin threaten rainfed agriculture, water resources, and ecosystem health.

The combined effects of short-term droughts, declining streamflow, and rising temperatures are expected to intensify pressures on food security, water resources, and ecosystem integrity, emphasizing Ethiopia’s acute sensitivity to climate variability [107–109]. To minimize the impacts of drought, an inclusive and integrated approach to adaptation and mitigation is essential to reduce the adverse effects of increasing dryness in the coming decades.

## Conclusion

This study provides a comprehensive assessment of hydroclimatic variability and drought dynamics in the eastern escarpment of the Abaya-Chamo sub-basin in southern Ethiopia. The results reveal complex but consistent patterns of environmental changes across rainfall, temperature, streamflow, and drought indices over the multi-decadal study period. Trend analysis of rainfall from 1987 to 2019 (four stations) and from 1990 to 2021 (two stations) indicated predominantly positive trends in annual precipitation; however, most were statistically insignificant, except at Hagereselam station, which exhibited a significant increasing trend (*p* < 0.05). Both maximum and minimum temperatures generally showed upward trends, with statistically significant increases in minimum temperatures observed at the Yirgachefe and Fisehagenet stations (P < 0.05). Streamflow analysis using the IHA framework revealed declining trends and increasing variability, particularly in low-flow conditions and flow reversals, indicating a shift toward more unstable hydrological regimes, likely influenced by both climate variability and anthropogenic factors such as land-use change and watershed degradation.

Drought assessment using the SPI and RDI_st_ at six- and twelve-month time scales revealed an increasing frequency of drought events since 1987. The SPI-6 analysis identified the most severe drought conditions at Arbaminch and Yirgachefe during 2010−2011, with index values of −2.41 and −3.31, respectively, while the RDI_st_-6 analysis indicated the highest frequency of drought occurrences at Arbaminch and Hagereselam. For the SPI-12 analysis, Hageremariam and Yirgachefe recorded the highest drought indices in 1996–1997 and 1994–1995 (−2.31 and −2.08, respectively). The RDI_st_-12 analysis showed that Yirgachefe (1996–1997) and Dilla (2002–2003) had the highest drought indices of −2.52 and **−**1.93, respectively. Shorter time scales (SPI-6 and RDI_st_-6) captured a higher frequency of drought events than longer time scales (SPI-12 and RDI_st_-12), highlighting the greater sensitivity of shorter accumulation periods in detecting drought onset and variability.

From both RDI_st_ indices showed greater sensitivity to hydroclimatic variability due to its incorporation of temperature effects through potential evapotranspiration. The concurrence of severe drought years across both indices reinforces the robustness of the findings and underscores the region’s vulnerability to prolonged dry spells. This multi-decadal assessment reveals increasing drought frequency, rising temperatures, and altered streamflow regimes in southern Ethiopia, reflecting the combined influences of local climatic variability and large-scale climate drivers such as El Niño southern Oscillation (ENSO). The findings provide a strong scientific basis for targeted drought mitigation, adaptive water resource management, and long-term climate- resilient planning in the Abaya-Chamo sub-basin. The Abaya Chamo sub-basin is of critical importance for Ethiopia’s agricultural productivity, water security, and tourism economy, particularly along the eastern escarpment. High -risk areas near Lake Abaya and Lake Chamo are lowland, hot, and drought-prone, reflecting the influence of elevation, land use, and annual variability.

Integrating climatic, hydrological, topographic, and socio-economic factors reveals compound risk zones that cannot be captured by single-variable indicators. Despite these contributions, the study is limited by factors related to data availability, station density, and the absence of land – use and future climate projection analyses, which may influence the spatial representation of hydroclimatic variability. Future research should integrate higher-resolution climate datasets, land-use change assessments, and climate model projections to better evaluate future drought risk. Including socio-economic vulnerability analyses would further support the development of effective, policy -relevant drought adaptation and resilience strategies for the Abaya -Chamo sub-basin. In conclusion, this study highlights the increasing vulnerability of the Abaya-Chamo sub-basin to hydroclimatic variability and drought, emphasizing the need for proactive, integrated, and multi- sectoral approaches to enhance resilience and ensure sustainable water and agricultural systems in southern Ethiopia.
