# Supplementary material for: Examination of hydro climatic trend and drought analysis for climate resilience in the eastern escarpment of Abaya Chamo sub-basin, Rift Valley Lake Basin, Ethiopia
Source: PLoS One. 2026 Aug 6;21(8):e0354913. doi: 10.1371/journal.pone.0354913 (PMC13446670; doi:10.1371/journal.pone.0354913)
Supplement: Supplementary file 1 [file pone.0354913.s004.pdf]

Reviewer comments to PONE-D-25-29739R3 "EXAMINATION OF HYDRO CLIMATIC TREND AND DROUGHT ANALYSIS FOR CLIMATE RESILIENCE IN THE EASTERN ESCARPMENT OF ABAYA CHAMO SUBBASIN, RIFT VALLEY LAKE BASIN, ETHIOPIA"
for: PLOS One

Summary

This manuscript is a revision from prior revisions. Since I did not see those revisions, I reviewed this as for a first submission; that ius .. I did not consider the authors response to revisions, nor any prior submissions.

This paper describes original research in which climatic trends were analyzed from 1987-2019 climate data for the Abaya Region of south-west Ethiopia. Climatic data were used to calculate several drought indices that have relevance to agricultural production. Trends in climatic data are widely published, however, this paper has scientific interest since this region has not had such a comprehensive analysis. The authors compare a variety of drought indices in this highly significant region.

The paper does not include detail of how the climatic data were measured, other than these data were part of the national climate records for Ethiopia. Some climate data were omitted from the analysis that might have been affected by variation in the surrounding vegetation, or changed agricultural practices. The statistical analysis are described in detail, and appear to have been analyzed appropriately.

The paper is generally well presented, although a number of grammatical revisions are suggested (see attachment).

Page numbers are the pages in the 69-page PDF document.

Specific points

1) P7/69 Abstract Suggest: "Rainfall trends for 1987-2019 were largely insignificant across five monitoring stations, except at Hagereselam station, which exhibited an upward trend." Figure 2 does not show statistical significance, and Pr detail could be added to Fig. 2 to validate the statement in the Abstract.
   Drought is generally a unit-less phenomenon, although there are some quantitative measures; suggest "The most severe drought was recorded at Yirgachefe (index score -3.31) and Arbaminch (index score -3.27) stations."
   L15 of Abstract. Use past tense 'provided'
2) P8/69. L4. Delete 'dramatically'; this is a value-based term that doesn't have a quantitative basis.. suggest to delete 'dramatically'
   L17.. replace 'like', with 'such as'
3) P9/69 L5. Use 'provide', rather than 'provides'
   L12. Use past tense 'integrated', rather than 'integrates'

L32. Use plural, ‘have’, not ‘has’

4) P11/69 L3. Use past tense ‘was’, not ‘is’
   3rd paragraph. The first sentence is poor.. its not the ‘data’ that are tested, but ‘trends in the data’; Suggest: “For each data-set, time trends were categorized as fitting either a null or alternative hypothesis.”
5) P12/69 L5. Add ‘the’; suggest: “In the present study, the Sen’s slope estimator was applied…”
6) P23/69 L40. Use past tense ‘demonstrated’, not “demonstrates’,
7) P25/69 L15. Use past tense ‘were’, not ‘are’
   L22-23. Consider revising this sentence “Previous studies indicate that droughts every three to four years, reflecting a marked increase in drought frequency”. Your previous definition of drought is a deviation from climatic norm.. thus the same level of water deficit that might be considered drought in one region might not be a drought in another region. It’s a colloquial idea that drought might occur at greater frequency, but rather, the base line water deficit has moved.. and annual water deficit has become greater.. drought is essentially a 1 in 10 year phenomenon? Avoid confusing ‘drought’ (a deviation from ‘average’) with measures of water deficit (where the average is decreasing).
8) P36/69 Fig 4. Y axis spelling “temperature”
