# Supplementary material for: Examination of hydro climatic trend and drought analysis for climate resilience in the eastern escarpment of Abaya Chamo sub-basin, Rift Valley Lake Basin, Ethiopia
Source: PLoS One. 2026 Aug 6;21(8):e0354913. doi: 10.1371/journal.pone.0354913 (PMC13446670; doi:10.1371/journal.pone.0354913)
Supplement: Supplementary file 4 [file pone.0354913.s005.docx]

EXAMINATION OF HYDRO CLIMATIC TREND AND DROUGHT ANALYSIS FOR CLIMATE RESILIENCE IN THE EASTERN ESCARPMENT OF ABAYA CHAMO SUB-BASIN, RIFT VALLEY LAKE BASIN, ETHIOPIA

Dear Academic Editor and Reviewers,

We sincerely thank the Academic Editor and reviewers for the careful evaluation of our manuscript entitled: “Examination of hydroclimatic trend and drought Analysis for climate resilience in the eastern escarpment of the Abaya–Chamo sub-basin, Rift Valley Lake basin, Ethiopia”. We greatly appreciate your constructive and detailed comments, which have significantly improved the quality and scientific contribution of our manuscript. We have carefully addressed all remaining comments and revised the manuscript accordingly. Detailed responses to each comment are provided below. All updated parts are colored by “**BLUE”** color in revised manuscript and response. A full point-by-point reply is included following for each reviewer: -

**REVIEWER #2:**

1. The proposed index is not validated against any independent indicators (e.g., health outcomes, disease burden, or external exposure metrics). Please provide a quantitative or qualitative validation demonstrating that the index meaningfully represents exposure risk and adds information beyond existing metrics.

- **Well, acknowledged:** Thank you for these valuable comments. We used an indirect (qualitative) validation technique because there were few independent quantitative datasets (e.g., health outcomes or direct exposure measurements) in the Abaya-Chamo sub-basin. The indicator was evaluated against known environmental gradients (elevation, temperature, drought-prone locations, and proximity to water bodies), documented vulnerability hotspots in the Wolayta Zone and Sidama Region, and local observations of water stress and crop failure. The results are in strong spatial agreement with these separate lines of evidence. Furthermore, the index was compared to traditional indicators such as rainfall variability, vegetation condition, and soil moisture, showing its potential to capture compound exposure patterns and transitional risk zones than single-variable metrics are unable to identify. Also, we have strengthened the discussion section by adding a dedicated paragraph that links our findings to practical implications.

2. The weighting of individual pollutants in the index is currently heuristic and insufficiently justified. Please provide a stronger rationale for the chosen weights and/or perform a sensitivity analysis to demonstrate that the main results are robust to alternative weighting choices.

- **Response of Authors:** We appreciate this comment. The weighting scheme was designed to reflect the **relative contribution of climatic, hydrological, topographic, and socio-economic factors** to exposure risk, based on existing literature and regional context. To address this concern, we have now conducted a **sensitivity analysis**, testing alternative weighting scenarios (e.g., equal weights and adjusted weights). The results indicate that the overall spatial patterns of exposure risk remain consistent across different weighting schemes, demonstrating the **robustness of the index**. These results have been added to the revised manuscript.

3. Please clarify more explicitly what conceptual or analytical problem the proposed index solves that is not already addressed by existing single-pollutant or population-weighted exposure measures. This clarification is essential to justify the scientific contribution of the study.

- **Acknowledged**: Thank you for highlighting this important point. The proposed index addresses a key limitation of existing approaches, which typically rely on single-variable indicators (e.g., rainfall or vegetation indices) or simplified exposure measures. Such approaches often fail to capture the multi-dimensional and spatially heterogeneous nature of exposure risk, particularly in data-scarce regions. The proposed index integrates climatic, hydrological, topographic, and socio-economic factors into a unified framework, enabling of identification of compound high-risk zones, detection of moderate-risk transitional areas this provides added analytical value beyond conventional metrics, particularly for supporting localized decision-making and climate risk planning. The novelty and contribution of the index have now been clarified in the Introduction and discussion sections modestly.

4. The manuscript claims policy relevance, but this is not demonstrated in a concrete way. Please include a clearer discussion or example showing how the proposed index would lead to different or improved policy insights compared to existing indicators.

- **Well, acknowledged:** We agree with this concern and have revised the manuscript to clearly state the conceptual gap addressed by this study. The revised text now emphasizes that existing studies often treat meteorological and hydrological drought independently. There is limited understanding of how drought propagates across systems, particularly in data-scarce regions. The integration of SPI, RDI, and IHA provides a more comprehensive framework to capture these linkages. This clarification is now explicitly presented in the Introduction and in conclusion part.

5. Please revise Reference 78 as follows:
Dogar, M. M. A., & Almazroui, M. (2022). Revisiting the strong and weak ENSO teleconnection impacts using a high-resolution atmospheric model. Atmospheric Environment, 270, 118866. <https://doi.org/10.1016/j.atmosenv.2021.118866>

- **Well, acknowledged:** Thank you for the recommendation. The manuscript has been revised accordingly after a careful review.

6. Please revise the following sentence to incorporate the additional relevant references:
Current text:

“The observed trends likely result from combined natural variability and anthropogenic forcing, highlighting the need for integrated climate and water management strategies in southern Ethiopia [76], [78], [79].”

Revised text:
“The observed trends likely result from combined natural variability and anthropogenic forcing, highlighting the need for integrated climate and water management strategies in southern Ethiopia [76], [78], [79], Dogar et al., 2017a, b; 2019, 2025].”

Please also ensure that the following references are correctly added to the reference list and formatted according to journal style:
<https://doi.org/10.1007/s12040-017-0804-4>
<https://doi.org/10.1002/2017JD026783>
<https://doi.org/10.1016/j.gloplacha.2018.10.004>
<https://doi.org/10.1088/2515-7620/adc675>

- **Well, acknowledged:** Thank you for this valuable suggestion. We have carefully reviewed it and added the references to the manuscript in accordance with the journal’s style.

**Reviewer #5:**

Although the authors have made significant revisions to address the reviewers' comments, a few issues still need to be addressed before the manuscript can be accepted as a research article. In general, I believe the logical structure of the paper requires further refinement, and the overall readability is somewhat lacking. Additionally, there are a few typographical errors, and the quality of the English writing would benefit from careful revision, ideally with the assistance of a native English speaker.

- **Well, acknowledged**: We thank Reviewer for the constructive comments. I have carefully addressed each point in detail in close collaboration with my co-author. The manuscript has been revised to improve its logical structure, clarity, and overall readability. We have reorganized key sections to enhance the flow of ideas and strengthened the connections between them. In addition, all typographical errors have been corrected, and the English has been thoroughly edited to improve grammar, clarity, and academic style. We believe these revisions have fully addressed the reviewer concerns.

**Reviewer #4:** All comments have been addressed

- **Response of Authors:** We thank reviewer #4 for positive feedback and acknowledge that all previous comments have been addressed
