# Supplementary material for: Examination of hydro climatic trend and drought analysis for climate resilience in the eastern escarpment of Abaya Chamo sub-basin, Rift Valley Lake Basin, Ethiopia
Source: PLoS One. 2026 Aug 6;21(8):e0354913. doi: 10.1371/journal.pone.0354913 (PMC13446670; doi:10.1371/journal.pone.0354913)
Supplement: Supplementary file 5 [file pone.0354913.s002.docx]

**Reply to the Reviewers**

Dear Reviewers,

First of all, we would like to thank the reviewers for their detailed observation of the manuscript and constructive comments. The comments were very helpful in improving the manuscript. The responses are colored in BLUE in both this paper and all of the corrected parts of the revised manuscript.

**Reviewer #1:**

1. Editorial (language)

- **Accepted:**  Thank you for your comments. We carefully updated the manuscript to improve its English grammar and clarity. We addressed grammatical errors and modified numerous sentences to enhance readability.

2. Remove numbering from the subtitles and titles

- **Accepted**: Thank you for your suggestion. We have removed all numbering from section titles and subtitles as requested.

3. Remove the accept and Reject Ho from the table, it is not part of the analysis

- **Acknowledged:** Thank you for the insight. We removed the "Accept H0" and "Reject H0" phrases from the table and provided the statistical interpretation in the text, in accordance with standard procedures for demonstrating hypothesis testing.

4. The data values are evaluated as an order-time series, and all subsequent data values are likened to each data value. The time series x1, x2, x3, xn represents n data points at a certain probability level. H0 is rejected in favor of Ha if the absolute value of S equals or exceeds a specified value of Sα/2, where Sα/2 is the smallest S that has a probability less than α/2 of appearing in the absence of a trend. Note that if S > 0, then later observations in the time series tend to be larger than those that appear earlier in the time series, and it is an indicator of an increasing trend, while the reverse is true if S < 0. Mathematically, Mann-Kandell S is determined as:
Not clear ....

- **Well, acknowledged**: Thank you for pointing this out. We agree that the previous explanation was unclear and confusing. In response, we changed the section to include a more detailed and accurate description of the Mann-Kendall test. Specifically, now: Clearly explain the technique for comparing each data point to the accomplishing values in the time series and provide a formal mathematical expression for the Mann-Kendall statistic (S). Also, clarify the hypotheses examined, as well as how the S and Z statistics are evaluated. Lastly, we replace some ambiguous terms with standard statistical language.

5. ...Indicators of Hydrologic Alteration (IHA) is a statistical software tool developed by the US Nature Conservancy to examine the extent to which human activities have altered flow regimes. For this study, the streamflow gauging station in the eastern escarpment of the Abaya Chamo basin at the Tore gauging station was analyzed using IHA software for 24 years (1990–2014) ...mathematical formulas be written

- **Well, acknowledged**: We appreciate your thoughtful comment regarding to mathematical formula. The Indicators of Hydrologic Alteration (IHA) software is basically a statistical tool that computes a variety of flow regime metrics using time series data. These factors are classified into five groups: 1 magnitude of monthly water conditions, 2 magnitude and length of annual extreme flows, 3 timing of annual extreme water conditions, 4 frequency and duration of high and low pulses, and 5 rate and frequency of water condition changes. While the IHA program does not expressly need user-inputted formulas, we believe that giving the underlying mathematical principles employed by the software improves transparency. Accordingly, we have revised the manuscript to include the key descriptive formulas associated with each group of indicators, thereby clarifying the statistical basis of our analysis.

6. .... Drought indices are used to characterize the intensitye.... use appropriate tense ...it should be simple past tense

- **Acknowledged:** revised in the revised manuscript

7. Use EndNote/Mendley for citation

- **Author responses:** We used Mendeley throughout our manuscript for managing and inserting all citations and references.

8. The correct name of ...Annual rainfall trend analysis... is Annual rainfall amount trend analysis

- **Author responses:** Thank you for the observation. We have corrected the title to **"Annual rainfall amount trend analysis"** as suggested.

**Reviewer #2:**

 Dear Editor and Authors,

This manuscript examines multi-decadal trends in rainfall, temperature, streamflow, and drought conditions in the eastern escarpment of the Abaya Chamo sub-basin, Rift Valley Lake Basin, Ethiopia. The authors applied the Mann–Kendall trend test and Sen’s slope estimator to detect trends in climatic variables, used Indicators of Hydrologic Alteration (IHA) to assess changes in streamflow characteristics, and calculated drought indices (SPI and RDI) to characterize drought severity and frequency over several decades. The study aims to inform adaptive water management strategies and improve understanding of climate variability in this region.

Based on novelty standards, this paper does not meet the threshold for publication in PLOS ONE, which requires studies to offer originality or a significant contribution to the body of knowledge. Therefore, I suggest that the paper be resubmitted after addressing the following concerns. These questions may help the authors strengthen or reframe the work:

1. How does this study provide new insights beyond the existing research on climatic trends and drought in Ethiopian basins? Please clearly articulate what specific gap your study fills compared to prior work.

- **Author responses: -**Thank you for your insightful comments. We completely agree that expressing the novelty and contribution of our research is significant. While prior research has investigated climate trends and drought in multiple Ethiopian basins, our work provides distinct and significant insights in several critical ways: -
- **Target a data scarce and under studied region**

Unlike most previous studies, which focused on more accessible basins, our study focuses on the eastern escarpment of the Abaya-Chamo sub-basin, a key but underestimated site in the Rift Valley basin. This focused emphasis fills a geographical gap in the literature and adds additional regional details.

- **Application of IHA in a Novel context**

The application of the Indicators of Hydrologic Alteration (IHA) approach to analyze changes in streamflow patterns is a novel contribution to this subbasin. This method allows for the discovery of small but ecologically significant changes in flow patterns a topic rarely addressed in prior Ethiopian basin research.

- **Assessing Climate variability for sustainable water use**

This study uses a multi-decadal dataset (1990 to 2013) of streamflow, rainfall, temperature, and two drought indices (SPI and RDI) to capture meteorological and hydrological drought patterns in the subbasin. The findings provide useful insights for adaptive water resource management, as well as evidence-based guidance for strategic planning in climate-vulnerable regions.

We have revised the introduction and discussion parts of the manuscript to explicitly highlight these contributions, better distinguish our work from existing studies, and clearly articulate the gap our study addresses. We hope these revisions clarify the novelty and relevance of our research.

2. Can you integrate additional datasets or variables such as land use change, socioeconomic impacts, or agricultural productivity to contextualize the hydrologic trends in a more interdisciplinary and policy-relevant way?

- **Well, acknowledged**: Thank you for your insightful comment. This study examined long-term patterns in precipitation and temperature, as well as drought analysis utilizing SPI and RDI. While land use, socioeconomic factors, and agricultural production are crucial for understanding drought impacts, they were excluded due to data limitations and the scope of the investigation. we agree that including these variables would enhance the work policy relevance, and we intend to address them in future study.

3. Have you validated your drought indices (SPI and RDI) against observed drought impacts (e.g., crop losses, water shortages) in the region? Empirical validation could improve the robustness of your conclusions.

- **Author responses:** Thank you for your evaluation. SPI and RDI were utilized in this work based on long-term climate data, however there was no direct validation against recorded drought impacts such as agricultural losses or water shortages due to a lack of consistent impact data for the region. we recognize that empirical validation would strengthen the study, and I want to incorporate such data into future research to improve the robustness and application of the findings.

4. Why did you limit the trend analysis to Mann-Kendall and Sen’s slope? Could more advanced methods (e.g., Bayesian trend detection, climate modeling, or machine learning approaches) yield deeper or more nuanced insights?

- **Author responses:** Thank you for the insightful comment. The Mann-Kendall test and Sen’s slope estimator were selected for their robustness, non-parametric nature, and wide acceptance in hydroclimatic trend analysis. These methods are effective for detecting monotonic trends in non-normally distributed data. While more advanced approaches such as Bayesian methods, climate models, or machine learning can offer deeper insights, they were beyond the scope of this study. I appreciate the suggestion and will consider incorporating such methods in future work to enhance analytical depth.

5. Could you compare your results to regional or continental datasets (e.g., CHIRPS precipitation, CRU temperature) to test consistency and improve the generalizability of your findings?

- **Author responses:** Thank you for the recommendation. We used ground-based historical station information for trend detection because of its superior resolution and localized accuracy. While we did not specifically compare our results to regional or continental datasets like CHIRPS or CRU, we recognize that doing so could improve the spatial consistency and generalizability of our findings. We intend to include such datasets in future analyses to add to station-based observations to assess regional-scale agreement. Present study only considered base point from stations

6. How do internal and external forcing—including anthropogenic drivers and large-scale climate variability (e.g., ENSO, NAO, IOD, Indian monsoon)—influence hydroclimatic trends in your study area? Please consider citing relevant recent studies, for example:
o Dogar et al., 2017a; 2017b; 2023; 2024; 2025
o Dogar and Sato, 2018; 2019
o Dogar and Almazroui, 2022
(Links to these references are provided below, at the end, for your convenience.)

- **Author responses:** Thank you for your valuable feedback. We acknowledge that both internal and external climate forcing including anthropogenic variables as well as large-scale climate variability such as ENSO, IOD, NAO, and the Indian Summer Monsoon have a substantial impact on hydroclimatic trends in the Abaya-Chamo subbasin. These determinants influence the timing, intensity, and spatial distribution of rainfall and drought occurrences throughout the region. Historical drought years, such as 1991-1992, 1998-1999, and 2010-2011, coincide with major El Niño or IOD occurrences, demonstrating strong teleconnection implications. And we incorporate some points in present paper.

7. How might aerosols—particularly volcanic aerosols—affect hydroclimatic variability and drought in your study region? Please discuss this potential influence and cite relevant studies, such as:
o Dogar et al., 2017a; 2023; 2024; 2025
o Dogar and Sato, 2018; 2019

- **Well, acknowledged**: Thank you for bringing up this crucial subject. Volcanic aerosols can have a substantial impact on hydroclimatic variability by changing air circulation, decreasing precipitation, and affecting surface temperatures. Depending on the timing and magnitude of eruptions, these effects can either exacerbate or alleviate drought conditions. Although our current study does not explicitly account for aerosol forcing, we acknowledge that volcanic aerosols, along with anthropogenic emissions, can influence regional rainfall and drought patterns. We will consider incorporating aerosol-related influences into future studies.

8. In Figure 6, what do “Series 1” and “Series 2” represent? Please define these clearly in the figure caption and methods.

- **Acknowledged**: Thank you for pointing this out. In Figure 6, Series 1represents the SPI-6 values determined for the first half of the hydrologic year (October-March), while Series 2 corresponds to the second half of the year (April-September). These two series were used to measure and compare the seasonal drought variations within each year. We modified both the figure caption and the methods section to clarify and interpret these phrases as follows: Series 1 (October to March) and 'Series 2 (April to September) are illustrated in figure 6 and each figure.

9. The time periods used for rainfall and temperature trend analyses start and end in different years (some from 1987, some from 1990, ending variously in 2019 or 2021). Please clarify the rationale for selecting different time ranges and explain whether this may affect the comparability of results across stations.

- **Accepted**: We appreciate your comment. The variation in starting and ending years between stations is caused by changes in the availability and completeness of historical climatic data at each location. Some stations have accurate records that began in 1990, but others had useable data as early as 1987. Similarly, end years differ based on the most recent validated data available for each location. Although there may be modest discrepancies in temporal coverage, we ensured that the periods selected for trend analysis were long enough (greater 30 years) to capture substantial climate trends while maintaining analytical consistency. It does not affect because each have more than two decades and enough for comparisons
