# Supplementary material for: Examination of hydro climatic trend and drought analysis for climate resilience in the eastern escarpment of Abaya Chamo sub-basin, Rift Valley Lake Basin, Ethiopia
Source: PLoS One. 2026 Aug 6;21(8):e0354913. doi: 10.1371/journal.pone.0354913 (PMC13446670; doi:10.1371/journal.pone.0354913)
Supplement: Supplementary file 6 [file pone.0354913.s003.docx]

Dear Academic Editor and Reviewers,

We sincerely thank the Academic Editor and reviewers for your careful evaluation of our manuscript entitled **“Examination of Hydro Climatic Trend and Drought Analysis for Climate Resilience in the Eastern Escarpment of Abaya Chamo Sub-Basin, Rift Valley Lake Basin, Ethiopia.”** We appreciate the constructive and detailed comments, which have helped us substantially improve the quality, and scientific contribution of the manuscript. We hope that the revisions satisfactorily address all comments.

**#Academic Editor Comment:** The manuscript still requires major revision, and a detailed response to all comments is needed.

- **Acknowledged:** We have carefully addressed all comments raised. The manuscript has been significantly updated, including the correction and inclusion of all suggested references, clear clarification of the study's novelty, deeper discussion with linking hydroclimatic trends to regional socioeconomic and water-resource implications, and serious language editing. All updated parts are colored by “**BLUE”** color in revised manuscript. A full point-by-point reply is included following for each reviewer: -

**Reviewer #2**

**Comment 1**: References 76–79 are not properly formatted; author names are incomplete, and references 78 and 79 appear duplicated. Suggested references were not adequately integrated, particularly regarding volcanism, ENSO, NAO, and the Indian monsoon.

- **Well, acknowledged:** We value the reviewer highlighting up these issues. All references number 76-79 have now been fully revised in accordance with PLOS ONE reference formatting guidelines. The duplicated references (formerly num: 78 and 79) have been eliminated, and the numbering has been updated correspondingly.

**Comment 2:** The novelty of the study is not clearly highlighted.

- **Acknowledge:** We modified the Introduction and Discussion sections to better convey the study's uniqueness. We now emphasize that this work: focuses on a highly data-scarce but climate-vulnerable sub-basin within the Ethiopian Rift Valley Lake Basin; integrates long-term hydroclimatic trend analysis with drought indices (SPI, and RDI), and IHA at multiple time scales; and provides one of the first basin-scale assessments linking drought dynamics to climate resilience planning in the Basin. These ideas are now explicitly articulated in the updated manuscript, emphasizing the uniqueness of our study.

**Comment 3**: The manuscript still contains grammatical and language issues.

- **Authors response:** The entire manuscript has been carefully proofread and edited for grammar, clarity, and academic tone. Redundant phrasing was removed, sentence structure improved, and terminology standardized throughout the text. We believe the revised version now meets PLOS ONE’s language requirements.

**Reviewer #3**

**Comment 1:** The claimed novelty (data-scarce region, use of IHA) is not sufficiently articulated; the manuscript reads as a general hydroclimatic study.

- **Well, acknowledged**: We appreciate this comment and have strengthened the framing of the study’s contribution. The Introduction now explicitly identifies the geographic, data, and methodological gaps addressed by this research. The Discussion further explains how applying IHA and drought indices in a data-scarce Rift Valley sub-basin contributes to climate-resilient water management planning.

**Comment 2:** The study is largely descriptive; stronger links to land use, agriculture, or socioeconomic impacts are needed.

- **Authors response:** We are added a dedicated paragraph in the Discussion section linking observed hydroclimatic and drought trends to known regional challenges, including smallholder rain fed and irrigated agriculture, increasing water demand, ecosystem stress, and livelihood vulnerability in the Abaya–Chamo basin.

**Comment 3**: SPI and RDI are not validated against observed impacts (e.g., crop loss, water shortages).

- Well, acknowledge: this limitation more explicitly in the revised manuscript. A new paragraph in the Discussion now clarifies that drought indices were not directly validated against observed impact data due to data limitations. We also outline how future research could integrate remote sensing products (e.g., NDVI, soil moisture) and local survey data to improve impact-based validation.

**Comment 4:** Different start and end years for datasets may affect trend comparability.

- **Authors Response:** We agree that this is an important consideration. The revised manuscript now clearly acknowledges this limitation. Additionally, we clarify that trend interpretations are made cautiously, emphasizing consistency rather than direct magnitude comparison across datasets.

**Comment 5: Reviewer-recommended Dogar et al. studies were not properly integrated.**

- **Well acknowledged Response:** All recommended Dogar et al. research have now been entirely incorporated and assessed in the context of large-scale teleconnections, aerosol forcing, and ENSO-related hydroclimatic variability, with a focus on East African rainfall dynamics in the discussion sections.
