# Supplementary material for: Examination of hydro climatic trend and drought analysis for climate resilience in the eastern escarpment of Abaya Chamo sub-basin, Rift Valley Lake Basin, Ethiopia
Source: PLoS One. 2026 Aug 6;21(8):e0354913. doi: 10.1371/journal.pone.0354913 (PMC13446670; doi:10.1371/journal.pone.0354913)
Supplement: Supplementary file 7 [file pone.0354913.s006.docx]

EXAMINATION OF HYDRO CLIMATIC TREND AND DROUGHT ANALYSIS FOR CLIMATE RESILIENCE IN THE EASTERN ESCARPMENT OF ABAYA CHAMO SUB-BASIN, RIFT VALLEY LAKE BASIN, ETHIOPIA

Dear Academic Editor and Reviewers,

We sincerely thank the Academic Editor and reviewers for the careful evaluation of our manuscript entitled: “Examination of hydroclimatic trend and drought Analysis for climate resilience in the eastern escarpment of the Abaya–Chamo sub-basin, Rift Valley Lake basin, Ethiopia”. We greatly appreciate your constructive and detailed comments, which have significantly improved the quality and scientific contribution of our manuscript. We have carefully addressed all remaining comments and revised the manuscript accordingly. Detailed responses to each comment are provided below. All updated parts are colored by “**BLUE”** color in revised manuscript and response. A full point-by-point reply is included following for each reviewer: -

1. Comment# P7/69 Abstract Suggest: “Rainfall trends for 1987-2019 were largely insignificant across five monitoring stations, except at Hagereselam station, which exhibited an upward trend.” Figure 2 does not show statistical significance, and Pr detail could be added to Fig. 2 to validate the statement in the Abstract. Drought is generally a unit-less phenomenon, although there are some quantitative measures; suggest “The most severe drought was recorded at Yirgachefe (index score 3.31) and Arbaminch (index score -3.27) stations.” L15 of Abstract. Use past tense ‘provided’

- **Well, acknowledged:** Thank you for this valuable suggestion. The recommended wording has been incorporated into the Abstract. The statistical significance of the rainfall trends is presented in table 4 and discussed in detail in the results section based on the Mann-Kendall trend test, including the corresponding P-values and hypothesis testing. Therefore, figure 2 has been retained to illustrate the temporal rainfall trends and linear regression, while statistical evidence supporting these interpretations is provided in table 4. We believe that the combination of figure 2 and table 4 adequately supports the statement presented in the abstract. We also appreciate the reviewer constructive comment regarding the description of drought severity. Accordingly, the abstract has been revised to express drought severity using drought index scores, rather than implying that drought has physical units. The revised sentences now read: The most severe drought was recorded at Yirgachefe (index score -3.31) and Arbaminch (index score -3.27).

2. Comment# P8/69. L4. Delete ‘dramatically’; this is a value-based term that doesn’t have a quantitative basis... suggest to delete ‘dramatically’ L17... replace ‘like’, with ‘such as’

- **Acknowledged: Thank you for this constructive suggestion. The word “dramatically” has been removed to maintain a more objective and quantitative tone. In addition, the word” like” has been replaced with “such as” at the indicated location to improve academic style and precision.**

3. Comment# P9/69 L5. Use ‘provide’, rather than ‘provides’ L12. Use past tense ‘integrated’, rather than ‘integrates’ L32. Use plural, ‘have’, not ‘has’.

- Well, acknowledged: Thank you for these helpful suggestions. The manuscript has been revised accordingly.

4. Comment# P11/69 L3. Use past tense ‘was’, not ‘is’ 3rd paragraph. The first sentence is poor. it’s not the ‘data’ that are tested, but ‘trends in the data’; Suggest: “For each data-set, time trends were categorized as fitting either a null or alternative hypothesis.”

- **Acknowledged:** Thank you for your valuable comments. The sentence has been revised to improve clarity and grammatical accuracy by replacing “is” with the past tense “was”. In addition, the suggested correction has been incorporated by rephrasing the sentence to emphasize that trends, rather than raw data, are evaluated. The revised sentence now reads: “For each dataset, trends were evaluated under either the null or alternative hypothesis.

5. Comment#5 P12/69 L5. Add ‘the’; suggest: “In the present study, the Sen’s slope estimator was applied…”

- **Acknowledged:** Thank you for the suggestion. The recommended grammatical revision has been incorporated by adding “the” before “Sen’s slope estimator”

6. Comment#6 P23/69 L40. Use past tense ‘demonstrated’, not ‘‘demonstrates’,

- **Acknowledged:** Thank you for the suggestion. The verb “demonstrates” has been revised to “demonstrated” to ensure tense consistency in the presentation of the study results

7. Comment#7 P25/69 L15. Use past tense ‘were’, not ‘are’ L22-23. Consider revising this sentence “Previous studies indicate that droughts every three to four years, reflecting a marked increase in drought frequency”. Your previous definition of drought is a deviation from climatic norm.. thus the same level of water deficit that might be considered drought in one region might not be a drought in another region. It’s a colloquial idea that drought might occur at greater frequency, but rather, the base line water deficit has moved.. and annual water deficit has become greater.. drought is essentially a 1 in 10 year phenomenon? Avoid confusing ‘drought’ (a deviation from ‘average’) with measures of water deficit (where the average is decreasing).

- **Acknowledged:** Thank you for this detailed conceptual clarification. The sentence has been revised to avoid implying that drought itself is defined by increased frequency. Instead, it now emphasizes that recent decades are characterized by increasing hydroclimatic variability and higher water deficits relative to the long-term climatic norm, rather implying that drought itself has become more frequent, in line with the reviewer suggestion.

8. Comment#9 P36/69 Fig 4. Y axis spelling “temperature”

- **Well, acknowledged:** Thank you for this constructive comment. The spelling error in the Y- axis label of figure 4 has been corrected from “temperature” to temperature”
