# Supplementary material for: Examination of hydro climatic trend and drought analysis for climate resilience in the eastern escarpment of Abaya Chamo sub-basin, Rift Valley Lake Basin, Ethiopia
Source: PLoS One. 2026 Aug 6;21(8):e0354913. doi: 10.1371/journal.pone.0354913 (PMC13446670; doi:10.1371/journal.pone.0354913)
Supplement: Peer review history

# Peer review: Decision Letter 0

**DOI:** 10.1371/journal.pone.0354913.r001

**Authors:** Suresh Devaraj

1 Jul 2025

PONE-D-25-29739

EXAMINATION OF HYDRO CLIMATIC TREND AND DROUGHT ANALYSIS FOR CLIMATE RESILIENCE IN THE EASTERN ESCARPMENT OF ABAYA CHAMO SUB-BASIN, RIFT VALLEY LAKE BASIN, ETHIOPIA

PLOS ONE

Dear Dr. Shomre,

Thank you for submitting your manuscript to PLOS ONE. After careful consideration, we feel that it has merit but does not fully meet PLOS ONE’s publication criteria as it currently stands. Therefore, we invite you to submit a revised version of the manuscript that addresses the points raised during the review process.

==============================

**ACADEMIC EDITOR:**

Given the extent of revisions required, we are recommending a Revision. If the concerns are satisfactorily addressed, we will proceed with a further round of review.

The reviewers have suggested some studies carried out for reference. Please make sure to refer the article and it is not mandatory to cite the same in the manuscript.

==============================

Please submit your revised manuscript by Aug 15 2025 11:59PM. If you will need more time than this to complete your revisions, please reply to this message or contact the journal office at plosone@plos.org. When you're ready to submit your revision, log on to https://www.editorialmanager.com/pone/ and select the 'Submissions Needing Revision' folder to locate your manuscript file.

Please include the following items when submitting your revised manuscript:

- A rebuttal letter that responds to each point raised by the academic editor and reviewer(s). You should upload this letter as a separate file labeled 'Response to Reviewers'.
- A marked-up copy of your manuscript that highlights changes made to the original version. You should upload this as a separate file labeled 'Revised Manuscript with Track Changes'.
- An unmarked version of your revised paper without tracked changes. You should upload this as a separate file labeled 'Manuscript'.

If you would like to make changes to your financial disclosure, please include your updated statement in your cover letter. Guidelines for resubmitting your figure files are available below the reviewer comments at the end of this letter.

If applicable, we recommend that you deposit your laboratory protocols in protocols.io to enhance the reproducibility of your results. Protocols.io assigns your protocol its own identifier (DOI) so that it can be cited independently in the future. For instructions see: https://journals.plos.org/plosone/s/submission-guidelines#loc-laboratory-protocols. Additionally, PLOS ONE offers an option for publishing peer-reviewed Lab Protocol articles, which describe protocols hosted on protocols.io. Read more information on sharing protocols at https://plos.org/protocols?utm_medium=editorial-email&utm_source=authorletters&utm_campaign=protocols.

We look forward to receiving your revised manuscript.

Kind regards,

Suresh Devaraj

Academic Editor

PLOS ONE

Journal Requirements:

When submitting your revision, we need you to address these additional requirements.

1. Please ensure that your manuscript meets PLOS ONE's style requirements, including those for file naming. The PLOS ONE style templates can be found at https://journals.plos.org/plosone/s/file?id=wjVg/PLOSOne_formatting_sample_main_body.pdf and https://journals.plos.org/plosone/s/file?id=ba62/PLOSOne_formatting_sample_title_authors_affiliations.pdf

2. Please note that PLOS One has specific guidelines on code sharing for submissions in which author-generated code underpins the findings in the manuscript. In these cases, we expect all author-generated code to be made available without restrictions upon publication of the work. Please review our guidelines at https://journals.plos.org/plosone/s/materials-and-software-sharing#loc-sharing-code and ensure that your code is shared in a way that follows best practice and facilitates reproducibility and reuse.

3. Please amend the manuscript submission data (via Edit Submission) to include author Muse Wldmchel.

4. Please amend your authorship list in your manuscript file to include author Muse Shomre.

5. Please amend your list of authors on the manuscript to ensure that each author is linked to an affiliation. Authors’ affiliations should reflect the institution where the work was done (if authors moved subsequently, you can also list the new affiliation stating “current affiliation:….” as necessary).

6. We note that Figure 1 in your submission contain [map/satellite] images which may be copyrighted. All PLOS content is published under the Creative Commons Attribution License (CC BY 4.0), which means that the manuscript, images, and Supporting Information files will be freely available online, and any third party is permitted to access, download, copy, distribute, and use these materials in any way, even commercially, with proper attribution. For these reasons, we cannot publish previously copyrighted maps or satellite images created using proprietary data, such as Google software (Google Maps, Street View, and Earth). For more information, see our copyright guidelines: http://journals.plos.org/plosone/s/licenses-and-copyright.

We require you to either (1) present written permission from the copyright holder to publish these figures specifically under the CC BY 4.0 license, or (2) remove the figures from your submission:

a. You may seek permission from the original copyright holder of Figure 1 to publish the content specifically under the CC BY 4.0 license.

We recommend that you contact the original copyright holder with the Content Permission Form (http://journals.plos.org/plosone/s/file?id=7c09/content-permission-form.pdf) and the following text:

“I request permission for the open-access journal PLOS ONE to publish XXX under the Creative Commons Attribution License (CCAL) CC BY 4.0 (http://creativecommons.org/licenses/by/4.0/). Please be aware that this license allows unrestricted use and distribution, even commercially, by third parties. Please reply and provide explicit written permission to publish XXX under a CC BY license and complete the attached form.”

Please upload the completed Content Permission Form or other proof of granted permissions as an "Other" file with your submission.

In the figure caption of the copyrighted figure, please include the following text: “Reprinted from [ref] under a CC BY license, with permission from [name of publisher], original copyright [original copyright year].”

b. If you are unable to obtain permission from the original copyright holder to publish these figures under the CC BY 4.0 license or if the copyright holder’s requirements are incompatible with the CC BY 4.0 license, please either i) remove the figure or ii) supply a replacement figure that complies with the CC BY 4.0 license. Please check copyright information on all replacement figures and update the figure caption with source information. If applicable, please specify in the figure caption text when a figure is similar but not identical to the original image and is therefore for illustrative purposes only.

The following resources for replacing copyrighted map figures may be helpful:

USGS National Map Viewer (public domain): http://viewer.nationalmap.gov/viewer/

The Gateway to Astronaut Photography of Earth (public domain): http://eol.jsc.nasa.gov/sseop/clickmap/

Maps at the CIA (public domain): https://www.cia.gov/library/publications/the-world-factbook/index.html and https://www.cia.gov/library/publications/cia-maps-publications/index.html

NASA Earth Observatory (public domain): http://earthobservatory.nasa.gov/

Landsat: http://landsat.visibleearth.nasa.gov/

USGS EROS (Earth Resources Observatory and Science (EROS) Center) (public domain): http://eros.usgs.gov/#

Natural Earth (public domain): http://www.naturalearthdata.com/

7. Please include captions for your Supporting Information files at the end of your manuscript, and update any in-text citations to match accordingly. Please see our Supporting Information guidelines for more information: http://journals.plos.org/plosone/s/supporting-information.

[Note: HTML markup is below. Please do not edit.]

Reviewers' comments:

Reviewer's Responses to Questions

**Comments to the Author**

1. Is the manuscript technically sound, and do the data support the conclusions?

The manuscript must describe a technically sound piece of scientific research with data that supports the conclusions. Experiments must have been conducted rigorously, with appropriate controls, replication, and sample sizes. The conclusions must be drawn appropriately based on the data presented.

Reviewer #1: Partly

Reviewer #2: Partly

**********

2. Has the statistical analysis been performed appropriately and rigorously?

Reviewer #1: Yes

Reviewer #2: Yes

**********

3. Have the authors made all data underlying the findings in their manuscript fully available?

The PLOS Data policy requires authors to make all data underlying the findings described in their manuscript fully available without restriction, with rare exception (please refer to the Data Availability Statement in the manuscript PDF file). The data should be provided as part of the manuscript or its supporting information, or deposited to a public repository. For example, in addition to summary statistics, the data points behind means, medians and variance measures should be available. If there are restrictions on publicly sharing data—e.g. participant privacy or use of data from a third party—those must be specified.

Reviewer #1: Yes

Reviewer #2: No

**********

4. Is the manuscript presented in an intelligible fashion and written in standard English?

PLOS ONE does not copyedit accepted manuscripts, so the language in submitted articles must be clear, correct, and unambiguous. Any typographical or grammatical errors should be corrected at revision, so please note any specific errors here.

Reviewer #1: Yes

Reviewer #2: Yes

**********

5. Review Comments to the Author

Please use the space provided to explain your answers to the questions above. You may also include additional comments for the author, including concerns about dual publication, research ethics, or publication ethics. (Please upload your review as an attachment if it exceeds 20,000 characters)

Reviewer #1: 1. Editorial (language )

2. Remove numbering from the subtitles and titles

3. Remove the accept and Reject Ho from the table, it is not part of the analysis

4. The data values are evaluated as an order-time series, and all subsequent data values are likened to each data value.

The time series x1, x2, x3, xn represents n data points at a certain probability level. H0 is rejected in favor of Ha if the

absolute value of S equals or exceeds a specified value of Sα/2, where Sα/2 is the smallest S that has a probability less

than α/2 of appearing in the absence of a trend. Note that if S > 0, then later observations in the time series tend to be

larger than those that appear earlier in the time series, and it is an indicator of an increasing trend, while the reverse is

true if S < 0. Mathematically, Mann-Kandell S is determined as:

Not clear ....

5. ...Indicators of Hydrologic Alteration (IHA) is a statistical software tool developed by the US Nature Conservancy to

examine the extent to which human activities have altered flow regimes. For this study, the streamflow gauging station

in the eastern escarpment of the Abaya Chamo basin at the Tore gauging station was analyzed using IHA software for

24 years (1990–2014) ...mathematical formulas be written

6. .....Drought indices are used to characterize the intensity....use appropriate tense ...it should be simple past tense

7. Use EndNote/Mendley for citation

8. the correct name of ...Annual rainfall trend analysis... is Annual rainfall amount trend analysis

Reviewer #2: Dear Editor and Authors,

This manuscript examines multi-decadal trends in rainfall, temperature, streamflow, and drought conditions in the eastern escarpment of the Abaya Chamo sub-basin, Rift Valley Lake Basin, Ethiopia. The authors applied the Mann–Kendall trend test and Sen’s slope estimator to detect trends in climatic variables, used Indicators of Hydrologic Alteration (IHA) to assess changes in streamflow characteristics, and calculated drought indices (SPI and RDI) to characterize drought severity and frequency over several decades. The study aims to inform adaptive water management strategies and improve understanding of climate variability in this region.

Based on novelty standards, this paper does not meet the threshold for publication in PLOS ONE, which requires studies to offer originality or a significant contribution to the body of knowledge. Therefore, I suggest that the paper be resubmitted after addressing the following concerns. These questions may help the authors strengthen or reframe the work:

1. How does this study provide new insights beyond the existing research on climatic trends and drought in Ethiopian basins? Please clearly articulate what specific gap your study fills compared to prior work.

2. Can you integrate additional datasets or variables—such as land use change, socioeconomic impacts, or agricultural productivity—to contextualize the hydrologic trends in a more interdisciplinary and policy-relevant way?

3. Have you validated your drought indices (SPI and RDI) against observed drought impacts (e.g., crop losses, water shortages) in the region? Empirical validation could improve the robustness of your conclusions.

4. Why did you limit the trend analysis to Mann-Kendall and Sen’s slope? Could more advanced methods (e.g., Bayesian trend detection, climate modeling, or machine learning approaches) yield deeper or more nuanced insights?

5. Could you compare your results to regional or continental datasets (e.g., CHIRPS precipitation, CRU temperature) to test consistency and improve the generalizability of your findings?

6. How do internal and external forcings—including anthropogenic drivers and large-scale climate variability (e.g., ENSO, NAO, IOD, Indian monsoon)—influence hydroclimatic trends in your study area? Please consider citing relevant recent studies, for example:

o Dogar et al., 2017a; 2017b; 2023; 2024; 2025

o Dogar and Sato, 2018; 2019

o Dogar and Almazroui, 2022

(Links to these references are provided below, at the end, for your convenience.)

7. How might aerosols—particularly volcanic aerosols—affect hydroclimatic variability and drought in your study region? Please discuss this potential influence and cite relevant studies, such as:

o Dogar et al., 2017a; 2023; 2024; 2025

o Dogar and Sato, 2018; 2019

8. In Figure 6, what do “Series 1” and “Series 2” represent? Please define these clearly in the figure caption and methods.

9. The time periods used for rainfall and temperature trend analyses start and end in different years (some from 1987, some from 1990, ending variously in 2019 or 2021). Please clarify the rationale for selecting different time ranges and explain whether this may affect the comparability of results across stations.

Suggested references:

• https://agupubs.onlinelibrary.wiley.com/doi/full/10.1002/2017JD026783

• https://link.springer.com/article/10.1007/s41748-022-00331-z

• https://agupubs.onlinelibrary.wiley.com/doi/full/10.1029/2023GL106114

• https://iopscience.iop.org/article/10.1088/2515-7620/adc675/meta

• https://agupubs.onlinelibrary.wiley.com/doi/full/10.1029/2018JD029003

• https://agupubs.onlinelibrary.wiley.com/doi/full/10.1029/2019JD030358

• https://link.springer.com/article/10.1007/s12040-017-0804-4

• https://www.sciencedirect.com/science/article/pii/S0921818117305982

• https://www.sciencedirect.com/science/article/pii/S1352231021006889

• https://juniperpublishers.com/jojha/pdf/JOJHA.MS.ID.555584.pdf

**********

6. PLOS authors have the option to publish the peer review history of their article (what does this mean?). If published, this will include your full peer review and any attached files.

If you choose “no”, your identity will remain anonymous but your review may still be made public.

**Do you want your identity to be public for this peer review?**  For information about this choice, including consent withdrawal, please see our Privacy Policy.

Reviewer #1: No

Reviewer #2: No

**********

[NOTE: If reviewer comments were submitted as an attachment file, they will be attached to this email and accessible via the submission site. Please log into your account, locate the manuscript record, and check for the action link "View Attachments". If this link does not appear, there are no attachment files.]

While revising your submission, please upload your figure files to the Preflight Analysis and Conversion Engine (PACE) digital diagnostic tool, https://pacev2.apexcovantage.com/. PACE helps ensure that figures meet PLOS requirements. To use PACE, you must first register as a user. Registration is free. Then, login and navigate to the UPLOAD tab, where you will find detailed instructions on how to use the tool. If you encounter any issues or have any questions when using PACE, please email PLOS at figures@plos.org. Please note that Supporting Information files do not need this step.

# Author comment: Author response to Decision Letter 1

**DOI:** 10.1371/journal.pone.0354913.r002

15 Sep 2025

Reply to the Reviewers

Dear Reviewers,

First of all, we would like to thank the reviewers for their detailed observation of the manuscript and constructive comments. The comments were very helpful in improving the manuscript. The responses are colored in BLUE in both this paper and all of the corrected parts of the revised manuscript.

Reviewer #1:

1. Editorial (language)

� Accepted: Thank you for your comments. We carefully updated the manuscript to improve its English grammar and clarity. We addressed grammatical errors and modified numerous sentences to enhance readability.

2. Remove numbering from the subtitles and titles

� Accepted: Thank you for your suggestion. We have removed all numbering from section titles and subtitles as requested.

3. Remove the accept and Reject Ho from the table, it is not part of the analysis

� Acknowledged: Thank you for the insight. We removed the "Accept H0" and "Reject H0" phrases from the table and provided the statistical interpretation in the text, in accordance with standard procedures for demonstrating hypothesis testing.

4. The data values are evaluated as an order-time series, and all subsequent data values are likened to each data value. The time series x1, x2, x3, xn represents n data points at a certain probability level. H0 is rejected in favor of Ha if the absolute value of S equals or exceeds a specified value of Sα/2, where Sα/2 is the smallest S that has a probability less than α/2 of appearing in the absence of a trend. Note that if S > 0, then later observations in the time series tend to be larger than those that appear earlier in the time series, and it is an indicator of an increasing trend, while the reverse is true if S < 0. Mathematically, Mann-Kandell S is determined as:

Not clear ....

� Well, acknowledged: Thank you for pointing this out. We agree that the previous explanation was unclear and confusing. In response, we changed the section to include a more detailed and accurate description of the Mann-Kendall test. Specifically, now: Clearly explain the technique for comparing each data point to the accomplishing values in the time series and provide a formal mathematical expression for the Mann-Kendall statistic (S). Also, clarify the hypotheses examined, as well as how the S and Z statistics are evaluated. Lastly, we replace some ambiguous terms with standard statistical language.

5. ...Indicators of Hydrologic Alteration (IHA) is a statistical software tool developed by the US Nature Conservancy to examine the extent to which human activities have altered flow regimes. For this study, the streamflow gauging station in the eastern escarpment of the Abaya Chamo basin at the Tore gauging station was analyzed using IHA software for 24 years (1990–2014) ...mathematical formulas be written

� Well, acknowledged: We appreciate your thoughtful comment regarding to mathematical formula. The Indicators of Hydrologic Alteration (IHA) software is basically a statistical tool that computes a variety of flow regime metrics using time series data. These factors are classified into five groups: 1 magnitude of monthly water conditions, 2 magnitude and length of annual extreme flows, 3 timing of annual extreme water conditions, 4 frequency and duration of high and low pulses, and 5 rate and frequency of water condition changes. While the IHA program does not expressly need user-inputted formulas, we believe that giving the underlying mathematical principles employed by the software improves transparency. Accordingly, we have revised the manuscript to include the key descriptive formulas associated with each group of indicators, thereby clarifying the statistical basis of our analysis.

6. .... Drought indices are used to characterize the intensitye.... use appropriate tense ...it should be simple past tense

� Acknowledged: revised in the revised manuscript

7. Use EndNote/Mendley for citation

� Author responses: We used Mendeley throughout our manuscript for managing and inserting all citations and references.

8. The correct name of ...Annual rainfall trend analysis... is Annual rainfall amount trend analysis

� Author responses: Thank you for the observation. We have corrected the title to "Annual rainfall amount trend analysis" as suggested.

Reviewer #2:

Dear Editor and Authors,

This manuscript examines multi-decadal trends in rainfall, temperature, streamflow, and drought conditions in the eastern escarpment of the Abaya Chamo sub-basin, Rift Valley Lake Basin, Ethiopia. The authors applied the Mann–Kendall trend test and Sen’s slope estimator to detect trends in climatic variables, used Indicators of Hydrologic Alteration (IHA) to assess changes in streamflow characteristics, and calculated drought indices (SPI and RDI) to characterize drought severity and frequency over several decades. The study aims to inform adaptive water management strategies and improve understanding of climate variability in this region.

Based on novelty standards, this paper does not meet the threshold for publication in PLOS ONE, which requires studies to offer originality or a significant contribution to the body of knowledge. Therefore, I suggest that the paper be resubmitted after addressing the following concerns. These questions may help the authors strengthen or reframe the work:

1. How does this study provide new insights beyond the existing research on climatic trends and drought in Ethiopian basins? Please clearly articulate what specific gap your study fills compared to prior work.

� Author responses: -Thank you for your insightful comments. We completely agree that expressing the novelty and contribution of our research is significant. While prior research has investigated climate trends and drought in multiple Ethiopian basins, our work provides distinct and significant insights in several critical ways: -

Target a data scarce and under studied region

Unlike most previous studies, which focused on more accessible basins, our study focuses on the eastern escarpment of the Abaya-Chamo sub-basin, a key but underestimated site in the Rift Valley basin. This focused emphasis fills a geographical gap in the literature and adds additional regional details.

Application of IHA in a Novel context

The application of the Indicators of Hydrologic Alteration (IHA) approach to analyze changes in streamflow patterns is a novel contribution to this subbasin. This method allows for the discovery of small but ecologically significant changes in flow patterns a topic rarely addressed in prior Ethiopian basin research.

Assessing Climate variability for sustainable water use

This study uses a multi-decadal dataset (1990 to 2013) of streamflow, rainfall, temperature, and two drought indices (SPI and RDI) to capture meteorological and hydrological drought patterns in the subbasin. The findings provide useful insights for adaptive water resource management, as well as evidence-based guidance for strategic planning in climate-vulnerable regions.

We have revised the introduction and discussion parts of the manuscript to explicitly highlight these contributions, better distinguish our work from existing studies, and clearly articulate the gap our study addresses. We hope these revisions clarify the novelty and relevance of our research.

2. Can you integrate additional datasets or variables such as land use change, socioeconomic impacts, or agricultural productivity to contextualize the hydrologic trends in a more interdisciplinary and policy-relevant way?

� Well, acknowledged: Thank you for your insightful comment. This study examined long-term patterns in precipitation and temperature, as well as drought analysis utilizing SPI and RDI. While land use, socioeconomic factors, and agricultural production are crucial for understanding drought impacts, they were excluded due to data limitations and the scope of the investigation. we agree that including these variables would enhance the work policy relevance, and we intend to address them in future study.

3. Have you validated your drought indices (SPI and RDI) against observed drought impacts (e.g., crop losses, water shortages) in the region? Empirical validation could improve the robustness of your conclusions.

� Author responses: Thank you for your evaluation. SPI and RDI were utilized in this work based on long-term climate data, however there was no direct validation against recorded drought impacts such as agricultural losses or water shortages due to a lack of consistent impact data for the region. we recognize that empirical validation would strengthen the study, and I want to incorporate such data into future research to improve the robustness and application of the findings.

4. Why did you limit the trend analysis to Mann-Kendall and Sen’s slope? Could more advanced methods (e.g., Bayesian trend detection, climate modeling, or machine learning approaches) yield deeper or more nuanced insights?

� Author responses: Thank you for the insightful comment. The Mann-Kendall test and Sen’s slope estimator were selected for their robustness, non-parametric nature, and wide acceptance in hydroclimatic trend analysis. These methods are effective for detecting monotonic trends in non-normally distributed data. While more advanced approaches such as Bayesian methods, climate models, or machine learning can offer deeper insights, they were beyond the scope of this study. I appreciate the suggestion and will consider incorporating such methods in future work to enhance analytical depth.

5. Could you compare your results to regional or continental datasets (e.g., CHIRPS precipitation, CRU temperature) to test consistency and improve the generalizability of your findings?

� Author responses: Thank you for the recommendation. We used ground-based historical station information for trend detection because of its superior resolution and localized accuracy. While we did not specifically compare our results to regional or continental datasets like CHIRPS or CRU, we recognize that doing so could improve the spatial consistency and generalizability of our findings. We intend to include such datasets in future analyses to add to station-based observations to assess regional-scale agreement. Present study only considered base point from stations

6. How do internal and external forcing—including anthropogenic drivers and large-scale climate variability (e.g., ENSO, NAO, IOD, Indian monsoon)—influence hydroclimatic trends in your study area? Please consider citing relevant recent studies, for example:

o Dogar et al., 2017a; 2017b; 2023; 2024; 2025

o Dogar and Sato, 2018; 2019

o Dogar and Almazroui, 2022

(Links to these references are provided below, at the end, for your convenience.)

� Author responses: Thank you for your valuable feedback. We acknowledge that both internal and external climate forcing including anthropogenic variables as well as large-scale climate variability such as ENSO, IOD, NAO, and the Indian Summer Monsoon have a substantial impact on hydroclimatic trends in the Abaya-Chamo subbasin. These determinants influence the timing, intensity, and spatial distribution of rainfall and drought occurrences throughout the region. Historical drought years, such as 1991-1992, 1998-1999, and 2010-2011, coincide with major El Niño or IOD occurrences, demonstrating strong teleconnection implications. And we incorporate some points in present paper.

7. How might aerosols—particularly volcanic aerosols—affect hydroclimatic variability and drought in your study region? Please discuss this potential influence and cite relevant studies, such as:

o Dogar et al., 2017a; 2023; 2024; 2025

o Dogar and Sato, 2018; 2019

� Well, acknowledged: Thank you for bringing up this crucial subject. Volcanic aerosols can have a substantial impact on hydroclimatic variability by changing air circulation, decreasing precipitation, and affecting surface temperatures. Depending on the timing and magnitude of eruptions, these effects can either exacerbate or alleviate drought conditions. Although our current study does not explicitly account for aerosol forcing, we acknowledge that volcanic aerosols, along with anthropogenic emissions, can influence regional rainfall and drought patterns. We will consider incorporating aerosol-related influences into future studies.

8. In Figure 6, what do “Series 1” and “Series 2” represent? Please define these clearly in the figure caption and methods.

� Acknowledged: Thank you for pointing this out. In Figure 6, Series 1represents the SPI-6 values determined for the first half of the hydrologic year (October-March), while Series 2 corresponds to the second half of the year (April-September). These two series were used to measure and compare the seasonal drought variations within each year. We modified both the figure caption and the methods section to clarify and interpret these phrases as follows: Series 1 (October to March) and 'Series 2 (April to September) are illustrated in figure 6 and each figure.

9. The time periods used for rainfall and temperature trend analyses start and end in different years (some from 1987, some from 1990, ending variously in 2019 or 2021). Please clarify the rationale for selecting different time ranges and explain whether this may affect the comparability of results across stations.

� Accepted: We appreciate your comment. The variation in starting and ending years between stations is caused by changes in the availability and completeness of historical climatic data at each location. Some stations have accurate records that began in 1990, but others had useable data as early as 1987. Similarly, end years differ based on the most recent validated data available for each location. Although there may be modest discrepancies in temporal coverage, we ensured that the periods selected for trend analysis were long enough (greater 30 years) to capture substantial climate trends while maintaining analytical consistency. It does not affect because each have more than two decades and enough for comparisons

Attachment Submitted filename: comment.docx

# Peer review: Decision Letter 1

**DOI:** 10.1371/journal.pone.0354913.r003

**Authors:** Majid Niazkar

9 Dec 2025

<div>PONE-D-25-29739R1

EXAMINATION OF HYDRO CLIMATIC TREND AND DROUGHT ANALYSIS FOR CLIMATE RESILIENCE IN THE EASTERN ESCARPMENT OF ABAYA CHAMO SUB-BASIN, RIFT VALLEY LAKE BASIN, ETHIOPIA

PLOS One

Dear Dr. Shomre,

Thank you for submitting your manuscript to PLOS ONE. After careful consideration, we feel that it has merit but does not fully meet PLOS ONE’s publication criteria as it currently stands. Therefore, we invite you to submit a revised version of the manuscript that addresses the points raised during the review process.

==============================

**ACADEMIC EDITOR:**  The manuscript still requires a major revision. The authors are advsied to provide a detailed response file to all comments.

==============================

Please submit your revised manuscript by Jan 23 2026 11:59PM. If you will need more time than this to complete your revisions, please reply to this message or contact the journal office at plosone@plos.org. When you're ready to submit your revision, log on to https://www.editorialmanager.com/pone/ and select the 'Submissions Needing Revision' folder to locate your manuscript file.

Please include the following items when submitting your revised manuscript:

- A rebuttal letter that responds to each point raised by the academic editor and reviewer(s). You should upload this letter as a separate file labeled 'Response to Reviewers'.
- A marked-up copy of your manuscript that highlights changes made to the original version. You should upload this as a separate file labeled 'Revised Manuscript with Track Changes'.
- An unmarked version of your revised paper without tracked changes. You should upload this as a separate file labeled 'Manuscript'.

If you would like to make changes to your financial disclosure, please include your updated statement in your cover letter. Guidelines for resubmitting your figure files are available below the reviewer comments at the end of this letter.

If applicable, we recommend that you deposit your laboratory protocols in protocols.io to enhance the reproducibility of your results. Protocols.io assigns your protocol its own identifier (DOI) so that it can be cited independently in the future. For instructions see: https://journals.plos.org/plosone/s/submission-guidelines#loc-laboratory-protocols. Additionally, PLOS ONE offers an option for publishing peer-reviewed Lab Protocol articles, which describe protocols hosted on protocols.io. Read more information on sharing protocols at https://plos.org/protocols?utm_medium=editorial-email&utm_source=authorletters&utm_campaign=protocols.

We look forward to receiving your revised manuscript.

Kind regards,

Majid Niazkar, Ph.D.

Academic Editor

PLOS One

Journal Requirements:

If the reviewer comments include a recommendation to cite specific previously published works, please review and evaluate these publications to determine whether they are relevant and should be cited. There is no requirement to cite these works unless the editor has indicated otherwise.

[Note: HTML markup is below. Please do not edit.]

Reviewers' comments:

Reviewer's Responses to Questions

**Comments to the Author**

1. If the authors have adequately addressed your comments raised in a previous round of review and you feel that this manuscript is now acceptable for publication, you may indicate that here to bypass the “Comments to the Author” section, enter your conflict of interest statement in the “Confidential to Editor” section, and submit your "Accept" recommendation.

Reviewer #2: (No Response)

Reviewer #3: All comments have been addressed

**********

2. Is the manuscript technically sound, and do the data support the conclusions?

The manuscript must describe a technically sound piece of scientific research with data that supports the conclusions. Experiments must have been conducted rigorously, with appropriate controls, replication, and sample sizes. The conclusions must be drawn appropriately based on the data presented.

Reviewer #2: Partly

Reviewer #3: Yes

**********

3. Has the statistical analysis been performed appropriately and rigorously?

Reviewer #2: N/A

Reviewer #3: Yes

**********

4. Have the authors made all data underlying the findings in their manuscript fully available?

The PLOS Data policy requires authors to make all data underlying the findings described in their manuscript fully available without restriction, with rare exception (please refer to the Data Availability Statement in the manuscript PDF file). The data should be provided as part of the manuscript or its supporting information, or deposited to a public repository. For example, in addition to summary statistics, the data points behind means, medians and variance measures should be available. If there are restrictions on publicly sharing data—e.g. participant privacy or use of data from a third party—those must be specified.

Reviewer #2: No

Reviewer #3: Yes

**********

5. Is the manuscript presented in an intelligible fashion and written in standard English?

PLOS ONE does not copyedit accepted manuscripts, so the language in submitted articles must be clear, correct, and unambiguous. Any typographical or grammatical errors should be corrected at revision, so please note any specific errors here.

Reviewer #2: No

Reviewer #3: Yes

**********

6. Review Comments to the Author

Please use the space provided to explain your answers to the questions above. You may also include additional comments for the author, including concerns about dual publication, research ethics, or publication ethics. (Please upload your review as an attachment if it exceeds 20,000 characters)

Reviewer #2: Dear Authors,

Thank you for submitting the revised version of your manuscript. I appreciate your efforts to address my earlier comments. However, several issues remain that need careful attention:

While you have attempted to incorporate my suggested references, they have not been adequately integrated into the manuscript. For instance, references numbered 76–79 are not formatted according to the journal’s reference style, and the author names are incomplete. Additionally, references 78 and 79 appear to be duplicates of the same source. Please correct these errors and ensure that all previously suggested references are properly included, particularly in the sections discussing volcanism, ENSO, NAO, and the role of the Indian monsoon.

I would also like to emphasize the need to clearly highlight the novelty of your study. As mentioned in my previous review, this aspect is crucial and should be made more explicit in the manuscript.

Finally, please carefully revise the manuscript to address the grammatical and language issues that are still present. A thorough proofreading would improve readability and clarity.

I encourage you to carefully consider these points in your next revision.

Best regards,While you have attempted to incorporate my suggested references, they have not been adequately integrated into the manuscript. For example, references numbered 76–79 are not formatted according to the journal’s reference style, and the author names are incomplete. Additionally, references 78 and 79 appear to be duplicates of the same source. Please correct these errors and ensure that all previously suggested references are properly included, particularly in the sections discussing volcanism, ENSO, NAO, and the role of the Indian monsoon. All of the references I suggested were relevant, so they should be included in full without being selectively omitted.

I would also like to emphasize the need to clearly highlight the novelty of your study. As mentioned in my previous review, this aspect is crucial and should be made more explicit in the manuscript.

Finally, please carefully revise the manuscript to address the remaining grammatical and language issues. A thorough proofreading will significantly improve the readability and clarity of the text.

Reviewer #3: hello

Although the authors claim novelty in focusing on a “data-scarce region” and using IHA, this is not strongly articulated in the manuscript itself. The introduction and discussion still read like a general hydroclimatic study rather than one filling a distinct geographic or methodological gap.

The study remains largely descriptive (trends in climate/hydrology). Reviewer #2 rightly asked for connections to land use, socioeconomics, or agriculture. The authors’ response (“future work”) is insufficient for publication in a high-impact journal. Even without new data, add a paragraph in the Discussion linking trends to known regional issues (e.g., smallholder irrigation vulnerability, tourism water demand) citing existing literature.

The authors admit SPI/RDI were not validated against observed impacts (crop loss, water shortage). This weakens the applied relevance. you can Acknowledge this limitation clearly in the Methods or Discussion and suggest how future studies could incorporate remote sensing (e.g., NDVI for vegetation stress) or local survey data.

The authors explain that data availability caused different start/end years, but they claim it “does not affect because each have more than two decades.” This is statistically questionable for trend comparison. authors can do the fallowing action as best : Conduct a sensitivity analysis for overlapping periods (e.g., 1990–2013) to confirm trend robustness, or clearly state this as a limitation.

Reviewer #2 specifically recommended citing Dogar et al. papers on ENSO, aerosols, and teleconnections. The authors responded but did not appear to integrate these citations into the manuscript.

**********

7. PLOS authors have the option to publish the peer review history of their article (what does this mean?). If published, this will include your full peer review and any attached files.

If you choose “no”, your identity will remain anonymous but your review may still be made public.

**Do you want your identity to be public for this peer review?**  For information about this choice, including consent withdrawal, please see our Privacy Policy.

Reviewer #2: No

Reviewer #3: No

**********

[NOTE: If reviewer comments were submitted as an attachment file, they will be attached to this email and accessible via the submission site. Please log into your account, locate the manuscript record, and check for the action link "View Attachments". If this link does not appear, there are no attachment files.]

To ensure your figures meet our technical requirements, please review our figure guidelines: https://journals.plos.org/plosone/s/figures

You may also use PLOS’s free figure tool, NAAS, to help you prepare publication quality figures: https://journals.plos.org/plosone/s/figures#loc-tools-for-figure-preparation.

NAAS will assess whether your figures meet our technical requirements by comparing each figure against our figure specifications.

# Author comment: Author response to Decision Letter 2

**DOI:** 10.1371/journal.pone.0354913.r004

6 Jan 2026

Dear Academic Editor and Reviewers,

We sincerely thank the Academic Editor and reviewers for your careful evaluation of our manuscript entitled “Examination of Hydro Climatic Trend and Drought Analysis for Climate Resilience in the Eastern Escarpment of Abaya Chamo Sub-Basin, Rift Valley Lake Basin, Ethiopia.” We appreciate the constructive and detailed comments, which have helped us substantially improve the quality, and scientific contribution of the manuscript. We hope that the revisions satisfactorily address all comments.

#Academic Editor Comment: The manuscript still requires major revision, and a detailed response to all comments is needed.

� Acknowledged: We have carefully addressed all comments raised. The manuscript has been significantly updated, including the correction and inclusion of all suggested references, clear clarification of the study's novelty, deeper discussion with linking hydroclimatic trends to regional socioeconomic and water-resource implications, and serious language editing. All updated parts are colored by “BLUE” color in revised manuscript. A full point-by-point reply is included following for each reviewer: -

Reviewer #2

Comment 1: References 76–79 are not properly formatted; author names are incomplete, and references 78 and 79 appear duplicated. Suggested references were not adequately integrated, particularly regarding volcanism, ENSO, NAO, and the Indian monsoon.

� Well, acknowledged: We value the reviewer highlighting up these issues. All references number 76-79 have now been fully revised in accordance with PLOS ONE reference formatting guidelines. The duplicated references (formerly num: 78 and 79) have been eliminated, and the numbering has been updated correspondingly.

Comment 2: The novelty of the study is not clearly highlighted.

� Acknowledge: We modified the Introduction and Discussion sections to better convey the study's uniqueness. We now emphasize that this work: focuses on a highly data-scarce but climate-vulnerable sub-basin within the Ethiopian Rift Valley Lake Basin; integrates long-term hydroclimatic trend analysis with drought indices (SPI, and RDI), and IHA at multiple time scales; and provides one of the first basin-scale assessments linking drought dynamics to climate resilience planning in the Basin. These ideas are now explicitly articulated in the updated manuscript, emphasizing the uniqueness of our study.

Comment 3: The manuscript still contains grammatical and language issues.

� Authors response: The entire manuscript has been carefully proofread and edited for grammar, clarity, and academic tone. Redundant phrasing was removed, sentence structure improved, and terminology standardized throughout the text. We believe the revised version now meets PLOS ONE’s language requirements.

Reviewer #3

Comment 1: The claimed novelty (data-scarce region, use of IHA) is not sufficiently articulated; the manuscript reads as a general hydroclimatic study.

� Well, acknowledged: We appreciate this comment and have strengthened the framing of the study’s contribution. The Introduction now explicitly identifies the geographic, data, and methodological gaps addressed by this research. The Discussion further explains how applying IHA and drought indices in a data-scarce Rift Valley sub-basin contributes to climate-resilient water management planning.

Comment 2: The study is largely descriptive; stronger links to land use, agriculture, or socioeconomic impacts are needed.

� Authors response: We are added a dedicated paragraph in the Discussion section linking observed hydroclimatic and drought trends to known regional challenges, including smallholder rain fed and irrigated agriculture, increasing water demand, ecosystem stress, and livelihood vulnerability in the Abaya–Chamo basin.

Comment 3: SPI and RDI are not validated against observed impacts (e.g., crop loss, water shortages).

� Well, acknowledge: this limitation more explicitly in the revised manuscript. A new paragraph in the Discussion now clarifies that drought indices were not directly validated against observed impact data due to data limitations. We also outline how future research could integrate remote sensing products (e.g., NDVI, soil moisture) and local survey data to improve impact-based validation.

Comment 4: Different start and end years for datasets may affect trend comparability.

� Authors Response: We agree that this is an important consideration. The revised manuscript now clearly acknowledges this limitation. Additionally, we clarify that trend interpretations are made cautiously, emphasizing consistency rather than direct magnitude comparison across datasets.

Comment 5: Reviewer-recommended Dogar et al. studies were not properly integrated.

� Well acknowledged Response: All recommended Dogar et al. research have now been entirely incorporated and assessed in the context of large-scale teleconnections, aerosol forcing, and ENSO-related hydroclimatic variability, with a focus on East African rainfall dynamics in the discussion sections.

Attachment Submitted filename: Dear Academic Editor and Reviewers.docx

# Peer review: Decision Letter 2

**DOI:** 10.1371/journal.pone.0354913.r005

**Authors:** Majid Niazkar

16 Mar 2026

PONE-D-25-29739R2

EXAMINATION OF HYDRO CLIMATIC TREND AND DROUGHT ANALYSIS FOR CLIMATE RESILIENCE IN THE EASTERN ESCARPMENT OF ABAYA CHAMO SUB-BASIN, RIFT VALLEY LAKE BASIN, ETHIOPIA

PLOS One

Dear Dr. Shomre,

Thank you for submitting your manuscript to PLOS ONE. After careful consideration, we feel that it has merit but does not fully meet PLOS ONE’s publication criteria as it currently stands. Therefore, we invite you to submit a revised version of the manuscript that addresses the points raised during the review process.

==============================

**ACADEMIC EDITOR:**  The manuscript needs a major revision. The authors do not need to cite any references suggested by the authors unless they are useful to enhance the manuscript quality. Please provide a point-by-point response to reviewers' comments to expedite the review process.

==============================

Please submit your revised manuscript by Apr 30 2026 11:59PM. If you will need more time than this to complete your revisions, please reply to this message or contact the journal office at plosone@plos.org. When you're ready to submit your revision, log on to https://www.editorialmanager.com/pone/ and select the 'Submissions Needing Revision' folder to locate your manuscript file.

Please include the following items when submitting your revised manuscript:

- A letter that responds to each point raised by the academic editor and reviewer(s). You should upload this letter as a separate file labeled 'Response to Reviewers'.
- A marked-up copy of your manuscript that highlights changes made to the original version. You should upload this as a separate file labeled 'Revised Manuscript with Track Changes'.
- An unmarked version of your revised paper without tracked changes. You should upload this as a separate file labeled 'Manuscript'.

If you would like to make changes to your financial disclosure, please include your updated statement in your cover letter. Guidelines for resubmitting your figure files are available below the reviewer comments at the end of this letter.

If applicable, we recommend that you deposit your laboratory protocols in protocols.io to enhance the reproducibility of your results. Protocols.io assigns your protocol its own identifier (DOI) so that it can be cited independently in the future. For instructions see: https://journals.plos.org/plosone/s/submission-guidelines#loc-laboratory-protocols. Additionally, PLOS ONE offers an option for publishing peer-reviewed Lab Protocol articles, which describe protocols hosted on protocols.io. Read more information on sharing protocols at https://plos.org/protocols?utm_medium=editorial-email&utm_source=authorletters&utm_campaign=protocols.

We look forward to receiving your revised manuscript.

Kind regards,

Majid Niazkar, Ph.D.

Academic Editor

PLOS One

Journal Requirements:

If the reviewer comments include a recommendation to cite specific previously published works, please review and evaluate these publications to determine whether they are relevant and should be cited. There is no requirement to cite these works unless the editor has indicated otherwise.

Additional Editor Comments:

The authors do not need to cite any references suggested by the authors unless they are useful to enhance the manuscript quality.

[Note: HTML markup is below. Please do not edit.]

Reviewers' comments:

Reviewer's Responses to Questions

**Comments to the Author**

1. If the authors have adequately addressed your comments raised in a previous round of review and you feel that this manuscript is now acceptable for publication, you may indicate that here to bypass the “Comments to the Author” section, enter your conflict of interest statement in the “Confidential to Editor” section, and submit your "Accept" recommendation.

Reviewer #2: (No Response)

Reviewer #4: All comments have been addressed

Reviewer #5: (No Response)

**********

2. Is the manuscript technically sound, and do the data support the conclusions?

The manuscript must describe a technically sound piece of scientific research with data that supports the conclusions. Experiments must have been conducted rigorously, with appropriate controls, replication, and sample sizes. The conclusions must be drawn appropriately based on the data presented.

Reviewer #2: Partly

Reviewer #4: Yes

Reviewer #5: (No Response)

**********

3. Has the statistical analysis been performed appropriately and rigorously?

Reviewer #2: No

Reviewer #4: Yes

Reviewer #5: (No Response)

**********

4. Have the authors made all data underlying the findings in their manuscript fully available?

The PLOS Data policy requires authors to make all data underlying the findings described in their manuscript fully available without restriction, with rare exception (please refer to the Data Availability Statement in the manuscript PDF file). The data should be provided as part of the manuscript or its supporting information, or deposited to a public repository. For example, in addition to summary statistics, the data points behind means, medians and variance measures should be available. If there are restrictions on publicly sharing data—e.g. participant privacy or use of data from a third party—those must be specified.

Reviewer #2: No

Reviewer #4: Yes

Reviewer #5: (No Response)

**********

5. Is the manuscript presented in an intelligible fashion and written in standard English?

PLOS ONE does not copyedit accepted manuscripts, so the language in submitted articles must be clear, correct, and unambiguous. Any typographical or grammatical errors should be corrected at revision, so please note any specific errors here.

Reviewer #2: No

Reviewer #4: Yes

Reviewer #5: (No Response)

**********

6. Review Comments to the Author

Please use the space provided to explain your answers to the questions above. You may also include additional comments for the author, including concerns about dual publication, research ethics, or publication ethics. (Please upload your review as an attachment if it exceeds 20,000 characters)

Reviewer #2: Decision: Major Revision

Dear Authors,

Thank you for submitting the revised version of your manuscript and for your detailed responses to the previous round of review. The revision has improved the clarity, structure, and overall presentation of the work, and the manuscript is now easier to follow and technically sound.

However, after further evaluation, we find that the study still requires substantial additional work before it can be considered for publication. While the proposed framework is coherent and the analysis is competently executed, the manuscript remains primarily a synthesis and reorganization of existing concepts rather than a clearly demonstrated methodological or conceptual advance. In its current form, the added scientific value and practical relevance of the proposed index are not yet sufficiently established.

We therefore invite you to address the following points in a further major revision:

1. The proposed index is not validated against any independent indicators (e.g., health outcomes, disease burden, or external exposure metrics). Please provide a quantitative or qualitative validation demonstrating that the index meaningfully represents exposure risk and adds information beyond existing metrics.

2. The weighting of individual pollutants in the index is currently heuristic and insufficiently justified. Please provide a stronger rationale for the chosen weights and/or perform a sensitivity analysis to demonstrate that the main results are robust to alternative weighting choices.

3. Please clarify more explicitly what conceptual or analytical problem the proposed index solves that is not already addressed by existing single-pollutant or population-weighted exposure measures. This clarification is essential to justify the scientific contribution of the study.

4. The manuscript claims policy relevance, but this is not demonstrated in a concrete way. Please include a clearer discussion or example showing how the proposed index would lead to different or improved policy insights compared to existing indicators.

5. Please revise Reference 78 as follows:

Dogar, M. M. A., & Almazroui, M. (2022). Revisiting the strong and weak ENSO teleconnection impacts using a high-resolution atmospheric model. Atmospheric Environment, 270, 118866. https://doi.org/10.1016/j.atmosenv.2021.118866

6. Please revise the following sentence to incorporate the additional relevant references:

Current text:

“The observed trends likely result from combined natural variability and anthropogenic forcing, highlighting the need for integrated climate and water management strategies in southern Ethiopia [76], [78], [79].”

Revised text:

“The observed trends likely result from combined natural variability and anthropogenic forcing, highlighting the need for integrated climate and water management strategies in southern Ethiopia [76], [78], [79], Dogar et al., 2017a, b; 2019, 2025].”

Please also ensure that the following references are correctly added to the reference list and formatted according to journal style:

https://doi.org/10.1007/s12040-017-0804-4

https://doi.org/10.1002/2017JD026783

https://doi.org/10.1016/j.gloplacha.2018.10.004

https://doi.org/10.1088/2515-7620/adc675

Addressing these points will substantially strengthen the manuscript by clarifying its scientific contribution, improving methodological robustness, and enhancing its relevance to the broader research and policy communities.

We therefore invite you to submit a major revision of the manuscript that responds thoroughly to the points above. Please include a detailed, point-by-point response explaining how each issue has been addressed.

I look forward to receiving your revised manuscript.

Reviewer #4: The author has thoroughly addressed all the comments and suggestions provided during the review process. As a reviewer, I acknowledge the author's efforts in rectifying the issues identified in the manuscript. For final suggestions, I recommend ensuring that all technical terms as fully abbreviations and accompanied by brief explanations to enhance the manuscript's accessibility for a broader audience. I extend my best wishes to the author for the success of their work.

Reviewer #5: Although the authors have made significant revisions to address the reviewers' comments, a few issues still need to be addressed before the manuscript can be accepted as a research article. In general, I believe the logical structure of the paper requires further refinement, and the overall readability is somewhat lacking. Additionally, there are a few typographical errors, and the quality of the English writing would benefit from careful revision, ideally with the assistance of a native English speaker.

**********

7. PLOS authors have the option to publish the peer review history of their article (what does this mean?). If published, this will include your full peer review and any attached files.

If you choose “no”, your identity will remain anonymous but your review may still be made public.

**Do you want your identity to be public for this peer review?**  For information about this choice, including consent withdrawal, please see our Privacy Policy.

Reviewer #2: No

Reviewer #4: **Yes:**  Md Abdullah Al Mamun

Reviewer #5: No

**********

[NOTE: If reviewer comments were submitted as an attachment file, they will be attached to this email and accessible via the submission site. Please log into your account, locate the manuscript record, and check for the action link "View Attachments". If this link does not appear, there are no attachment files.]

To ensure your figures meet our technical requirements, please review our figure guidelines: https://journals.plos.org/plosone/s/figures

You may also use PLOS’s free figure tool, NAAS, to help you prepare publication quality figures: https://journals.plos.org/plosone/s/figures#loc-tools-for-figure-preparation.

NAAS will assess whether your figures meet our technical requirements by comparing each figure against our figure specifications.

# Author comment: Author response to Decision Letter 3

**DOI:** 10.1371/journal.pone.0354913.r006

2 Apr 2026

Dear Editor,

We thank the Academic Editor and the reviewers for their valuable comments and suggestions. We have carefully revised the manuscript to address all concerns raised.

In particular, we have:

� Improved the statistical analysis and clarified the methodology

� Added validation and justification for the proposed index

� Strengthened the discussion on scientific contribution and policy relevance

� Revised the manuscript for clarity, structure, and English language

� Updated and corrected the references as suggested

A detailed, point-by-point response to all reviewer comments is provided in the “Response to Reviewers” document. We hope that the revised manuscript meets the requirements for publication in PLOS ONE.

Thank you for your consideration.

Attachment Submitted filename: Response to Reviewers.docx

# Peer review: Decision Letter 3

**DOI:** 10.1371/journal.pone.0354913.r007

**Authors:** Majid Niazkar

22 Jun 2026

PONE-D-25-29739R3

EXAMINATION OF HYDRO CLIMATIC TREND AND DROUGHT ANALYSIS FOR CLIMATE RESILIENCE IN THE EASTERN ESCARPMENT OF ABAYA CHAMO SUB-BASIN, RIFT VALLEY LAKE BASIN, ETHIOPIA

PLOS One

Dear Dr. Shomre,

Thank you for submitting your manuscript to PLOS ONE. After careful consideration, we feel that it has merit but does not fully meet PLOS ONE’s publication criteria as it currently stands. Therefore, we invite you to submit a revised version of the manuscript that addresses the points raised during the review process.

==============================

**ACADEMIC EDITOR:**  Based on the review assessment, the manuscript can be accepted after a minor revision.

==============================

Please submit your revised manuscript by Aug 06 2026 11:59PM. If you will need more time than this to complete your revisions, please reply to this message or contact the journal office at plosone@plos.org. When you're ready to submit your revision, log on to https://www.editorialmanager.com/pone/ and select the 'Submissions Needing Revision' folder to locate your manuscript file.

Please include the following items when submitting your revised manuscript:

- A letter that responds to each point raised by the academic editor and reviewer(s). You should upload this letter as a separate file labeled 'Response to Reviewers'.
- A marked-up copy of your manuscript that highlights changes made to the original version. You should upload this as a separate file labeled 'Revised Manuscript with Track Changes'.
- An unmarked version of your revised paper without tracked changes. You should upload this as a separate file labeled 'Manuscript'.

If you would like to make changes to your financial disclosure, please include your updated statement in your cover letter. Guidelines for resubmitting your figure files are available below the reviewer comments at the end of this letter.

If applicable, we recommend that you deposit your laboratory protocols in protocols.io to enhance the reproducibility of your results. Protocols.io assigns your protocol its own identifier (DOI) so that it can be cited independently in the future. For instructions see: https://journals.plos.org/plosone/s/submission-guidelines#loc-laboratory-protocols. Additionally, PLOS ONE offers an option for publishing peer-reviewed Lab Protocol articles, which describe protocols hosted on protocols.io. Read more information on sharing protocols at https://plos.org/protocols?utm_medium=editorial-email&utm_source=authorletters&utm_campaign=protocols.

As the corresponding author, your ORCID iD is verified in the submission system and will appear in the published article. PLOS supports the use of ORCID, and we encourage all coauthors to register for an ORCID iD and use it as well. Please encourage your coauthors to verify their ORCID iD within the submission system before final acceptance, as unverified ORCID iDs will not appear in the published article. *Only*  the individual author can complete the verification step; PLOS staff *cannot*  verify ORCID iDs on behalf of authors.

We look forward to receiving your revised manuscript.

Kind regards,

Majid Niazkar, Ph.D.

Academic Editor

PLOS One

**Journal Requirements:**

1. If the reviewer comments include a recommendation to cite specific previously published works, please review and evaluate these publications to determine whether they are relevant and should be cited. There is no requirement to cite these works unless the editor has indicated otherwise.

2. Please review your reference list to ensure that it is complete and correct. If you have cited papers that have been retracted, please include the rationale for doing so in the manuscript text, or remove these references and replace them with relevant current references. Any changes to the reference list should be mentioned in the rebuttal letter that accompanies your revised manuscript. If you need to cite a retracted article, indicate the article’s retracted status in the References list and also include a citation and full reference for the retraction notice.

**Additional Editor Comments:**

Based on the review assessment, the manuscript can be accepted after a minor revision.

[Note: HTML markup is below. Please do not edit.]

Reviewers' comments:

Reviewer's Responses to Questions

**Comments to the Author**

1. If the authors have adequately addressed your comments raised in a previous round of review and you feel that this manuscript is now acceptable for publication, you may indicate that here to bypass the “Comments to the Author” section, enter your conflict of interest statement in the “Confidential to Editor” section, and submit your "Accept" recommendation.

Reviewer #2: All comments have been addressed

Reviewer #6: All comments have been addressed

Reviewer #7: All comments have been addressed

**********

2. Is the manuscript technically sound, and do the data support the conclusions?

The manuscript must describe a technically sound piece of scientific research with data that supports the conclusions. Experiments must have been conducted rigorously, with appropriate controls, replication, and sample sizes. The conclusions must be drawn appropriately based on the data presented.

Reviewer #2: Partly

Reviewer #6: Yes

Reviewer #7: Yes

**********

3. Has the statistical analysis been performed appropriately and rigorously?

Reviewer #2: Yes

Reviewer #6: Yes

Reviewer #7: Yes

**********

4. Have the authors made all data underlying the findings in their manuscript fully available?

The PLOS Data policy requires authors to make all data underlying the findings described in their manuscript fully available without restriction, with rare exception (please refer to the Data Availability Statement in the manuscript PDF file). The data should be provided as part of the manuscript or its supporting information, or deposited to a public repository. For example, in addition to summary statistics, the data points behind means, medians and variance measures should be available. If there are restrictions on publicly sharing data—e.g. participant privacy or use of data from a third party—those must be specified.

Reviewer #2: No

Reviewer #6: (No Response)

Reviewer #7: Yes

**********

5. Is the manuscript presented in an intelligible fashion and written in standard English?

PLOS ONE does not copyedit accepted manuscripts, so the language in submitted articles must be clear, correct, and unambiguous. Any typographical or grammatical errors should be corrected at revision, so please note any specific errors here.

Reviewer #2: No

Reviewer #6: Yes

Reviewer #7: Yes

**********

6. Review Comments to the Author

Please use the space provided to explain your answers to the questions above. You may also include additional comments for the author, including concerns about dual publication, research ethics, or publication ethics. (Please upload your review as an attachment if it exceeds 20,000 characters)

Reviewer #2: (No Response)

Reviewer #6: MS No: PONE-D-25-29739R3_reviewer

MS Title: Examination of hydro climatic trend and drought analysis for Climate resilience in the eastern escarpment of Abaya Chamo sub Basin, Rift valley lake basin, Ethiopia

The MS is improved. The authors have addressed the concerns / quarries and clarifications required as per the previous reviewers. Hence, I recommend considering the paper for publication.

Reviewer #7: Reviewer comments to PONE-D-25-29739R3 “EXAMINATION OF HYDRO CLIMATIC TREND AND DROUGHT ANALYSIS FOR CLIMATE RESILIENCE IN THE EASTERN ESCARPMENT OF ABAYA CHAMO SUBBASIN, RIFT VALLEY LAKE BASIN, ETHIOPIA”

for: PLOS One

Summary

This manuscript is a revision from prior revisions. Since I did not see those revisions, I reviewed this as for a first submission; that is .. I did not consider the author’s response to revisions, nor any prior submissions.

This paper describes original research in which climatic trends were analyzed from 1987-2019 climate data for the Abaya Region of south-west Ethiopia. Climatic data were used to calculate several drought indices that have relevance to agricultural production. Trends in climatic data are widely published, however, this paper has scientific interest since this region has not had such a comprehensive analysis. The authors compare a variety of drought indices in this highly significant region.

The paper does not include detail of how the climatic data were measured, other than these data were part of the national climate records for Ethiopia. Some climate data were omitted from the analysis that might have been affected by variation in the surrounding vegetation, or changed agricultural practices. The statistical analysis are described in detail, and appear to have been analyzed appropriately.

The paper is generally well presented, although a number of grammatical revisions are suggested (see attachment).

Page numbers are the pages in the 69-page PDF document.

Specific points

1) P7/69 Abstract Suggest: “Rainfall trends for 1987-2019 were largely insignificant across five monitoring stations, except at Hagereselam station, which exhibited an upward trend.” Figure 2 does not show statistical significance, and Pr detail could be added to Fig. 2 to validate the statement in the Abstract.

Drought is generally a unit-less phenomenon, although there are some quantitative measures; suggest “The most severe drought was recorded at Yirgachefe (index score -3.31) and Arbaminch (index score -3.27) stations.”

L15 of Abstract. Use past tense ‘provided’

2) P8/69. L4. Delete ‘dramatically’; this is a value-based term that doesn’t have a quantitative basis.. suggest to delete ‘dramatically’

L17.. replace ‘like’, with ‘such as’

3) P9/69 L5. Use ‘provide’, rather than ‘provides’

L12. Use past tense ‘integrated’, rather than ‘integrates’

L32. Use plural, ‘have’, not ‘has’

4) P11/69 L3. Use past tense ‘was’, not ‘is’

3rd paragraph. The first sentence is poor.. its not the ‘data’ that are tested, but ‘trends in the data’; Suggest: “For each data-set, time trends were categorized as fitting either a null or alternative hypothesis.”

5) P12/69 L5. Add ‘the’; suggest: “In the present study, the Sen’s slope estimator was applied…”

6) P23/69 L40. Use past tense ‘demonstrated’, not ‘‘demonstrates’,

7) P25/69 L15. Use past tense ‘were’, not ‘are’

L22-23. Consider revising this sentence “Previous studies indicate that droughts every three to four years, reflecting a marked increase in drought frequency”. Your previous definition of drought is a deviation from climatic norm.. thus the same level of water deficit that might be considered drought in one region might not be a drought in another region. It’s a colloquial idea that drought might occur at greater frequency, but rather, the base line water deficit has moved.. and annual water deficit has become greater.. drought is essentially a 1 in 10 year phenomenon? Avoid confusing ‘drought’ (a deviation from ‘average’) with measures of water deficit (where the average is decreasing).

8) P36/69 Fig 4. Y axis spelling “temperature”

**********

7. PLOS authors have the option to publish the peer review history of their article (what does this mean?). If published, this will include your full peer review and any attached files.

If you choose “no”, your identity will remain anonymous but your review may still be made public.

**Do you want your identity to be public for this peer review?**  For information about this choice, including consent withdrawal, please see our Privacy Policy.

Reviewer #2: No

Reviewer #6: **Yes:**  Dr P A Azeez

Reviewer #7: No

**********

[NOTE: If reviewer comments were submitted as an attachment file, they will be attached to this email and accessible via the submission site. Please log into your account, locate the manuscript record, and check for the action link "View Attachments". If this link does not appear, there are no attachment files.]

To ensure your figures meet our technical requirements, please review our figure guidelines: https://journals.plos.org/plosone/s/figures

You may also use PLOS’s free figure tool, NAAS, to help you prepare publication quality figures: https://journals.plos.org/plosone/s/figures#loc-tools-for-figure-preparation.

NAAS will assess whether your figures meet our technical requirements by comparing each figure against our figure specifications.

Attachment Submitted filename: Review #316 PONE-D-25-29739R3.pdf

# Author comment: Author response to Decision Letter 4

**DOI:** 10.1371/journal.pone.0354913.r008

3 Jul 2026

EXAMINATION OF HYDRO CLIMATIC TREND AND DROUGHT ANALYSIS FOR CLIMATE RESILIENCE IN THE EASTERN ESCARPMENT OF ABAYA CHAMO SUB-BASIN, RIFT VALLEY LAKE BASIN, ETHIOPIA

Dear Academic Editor and Reviewers,

We sincerely thank the Academic Editor and reviewers for the careful evaluation of our manuscript entitled: “Examination of hydroclimatic trend and drought Analysis for climate resilience in the eastern escarpment of the Abaya–Chamo sub-basin, Rift Valley Lake basin, Ethiopia”. We greatly appreciate your constructive and detailed comments, which have significantly improved the quality and scientific contribution of our manuscript. We have carefully addressed all remaining comments and revised the manuscript accordingly. Detailed responses to each comment are provided below. All updated parts are colored by “BLUE” color in revised manuscript and response. A full point-by-point reply is included following for each reviewer: -

1. Comment# P7/69 Abstract Suggest: “Rainfall trends for 1987-2019 were largely insignificant across five monitoring stations, except at Hagereselam station, which exhibited an upward trend.” Figure 2 does not show statistical significance, and Pr detail could be added to Fig. 2 to validate the statement in the Abstract. Drought is generally a unit-less phenomenon, although there are some quantitative measures; suggest “The most severe drought was recorded at Yirgachefe (index score 3.31) and Arbaminch (index score -3.27) stations.” L15 of Abstract. Use past tense ‘provided’

� Well, acknowledged: Thank you for this valuable suggestion. The recommended wording has been incorporated into the Abstract. The statistical significance of the rainfall trends is presented in table 4 and discussed in detail in the results section based on the Mann-Kendall trend test, including the corresponding P-values and hypothesis testing. Therefore, figure 2 has been retained to illustrate the temporal rainfall trends and linear regression, while statistical evidence supporting these interpretations is provided in table 4. We believe that the combination of figure 2 and table 4 adequately supports the statement presented in the abstract. We also appreciate the reviewer constructive comment regarding the description of drought severity. Accordingly, the abstract has been revised to express drought severity using drought index scores, rather than implying that drought has physical units. The revised sentences now read: The most severe drought was recorded at Yirgachefe (index score -3.31) and Arbaminch (index score -3.27).

2. Comment# P8/69. L4. Delete ‘dramatically’; this is a value-based term that doesn’t have a quantitative basis... suggest to delete ‘dramatically’ L17... replace ‘like’, with ‘such as’

� Acknowledged: Thank you for this constructive suggestion. The word “dramatically” has been removed to maintain a more objective and quantitative tone. In addition, the word” like” has been replaced with “such as” at the indicated location to improve academic style and precision.

3. Comment# P9/69 L5. Use ‘provide’, rather than ‘provides’ L12. Use past tense ‘integrated’, rather than ‘integrates’ L32. Use plural, ‘have’, not ‘has’.

� Well, acknowledged: Thank you for these helpful suggestions. The manuscript has been revised accordingly.

4. Comment# P11/69 L3. Use past tense ‘was’, not ‘is’ 3rd paragraph. The first sentence is poor. it’s not the ‘data’ that are tested, but ‘trends in the data’; Suggest: “For each data-set, time trends were categorized as fitting either a null or alternative hypothesis.”

� Acknowledged: Thank you for your valuable comments. The sentence has been revised to improve clarity and grammatical accuracy by replacing “is” with the past tense “was”. In addition, the suggested correction has been incorporated by rephrasing the sentence to emphasize that trends, rather than raw data, are evaluated. The revised sentence now reads: “For each dataset, trends were evaluated under either the null or alternative hypothesis.

5. Comment#5 P12/69 L5. Add ‘the’; suggest: “In the present study, the Sen’s slope estimator was applied…”

� Acknowledged: Thank you for the suggestion. The recommended grammatical revision has been incorporated by adding “the” before “Sen’s slope estimator”

6. Comment#6 P23/69 L40. Use past tense ‘demonstrated’, not ‘‘demonstrates’,

� Acknowledged: Thank you for the suggestion. The verb “demonstrates” has been revised to “demonstrated” to ensure tense consistency in the presentation of the study results

7. Comment#7 P25/69 L15. Use past tense ‘were’, not ‘are’ L22-23. Consider revising this sentence “Previous studies indicate that droughts every three to four years, reflecting a marked increase in drought frequency”. Your previous definition of drought is a deviation from climatic norm.. thus the same level of water deficit that might be considered drought in one region might not be a drought in another region. It’s a colloquial idea that drought might occur at greater frequency, but rather, the base line water deficit has moved.. and annual water deficit has become greater.. drought is essentially a 1 in 10 year phenomenon? Avoid confusing ‘drought’ (a deviation from ‘average’) with measures of water deficit (where the average is decreasing).

� Acknowledged: Thank you for this detailed conceptual clarification. The sentence has been revised to avoid implying that drought itself is defined by increased frequency. Instead, it now emphasizes that recent decades are characterized by increasing hydroclimatic variability and higher water deficits relative to the long-term climatic norm, rather implying that drought itself has become more frequent, in line with the reviewer suggestion.

8. Comment#9 P36/69 Fig 4. Y axis spelling “temperature”

� Well, acknowledged: Thank you for this constructive comment. The spelling error in the Y- axis label of figure 4 has been corrected from “temperature” to temperature”

Attachment Submitted filename: Response_to_Reviewers_auresp_4.docx

# Editor report: Decision Letter 4

**DOI:** 10.1371/journal.pone.0354913.r009

**Authors:** Majid Niazkar

14 Jul 2026

EXAMINATION OF HYDRO CLIMATIC TREND AND DROUGHT ANALYSIS FOR CLIMATE RESILIENCE IN THE EASTERN ESCARPMENT OF ABAYA CHAMO SUB-BASIN, RIFT VALLEY LAKE BASIN, ETHIOPIA

PONE-D-25-29739R4

Dear Dr. Shomre,

We’re pleased to inform you that your manuscript has been judged scientifically suitable for publication and will be formally accepted for publication once it meets all outstanding technical requirements.

Within one week, you’ll receive an e-mail detailing the required amendments. When these have been addressed, you’ll receive a formal acceptance letter and your manuscript will be scheduled for publication.

An invoice will be generated when your article is formally accepted. Please note, if your institution has a publishing partnership with PLOS and your article meets the relevant criteria, all or part of your publication costs will be covered. Please make sure your user information is up-to-date by logging into Editorial Manager at Editorial Manager® and clicking the ‘Update My Information' link at the top of the page. For questions related to billing, please contact billing support.

If your institution or institutions have a press office, please notify them about your upcoming paper to help maximize its impact. If they’ll be preparing press materials, please inform our press team as soon as possible -- no later than 48 hours after receiving the formal acceptance. Your manuscript will remain under strict press embargo until 2 pm Eastern Time on the date of publication. For more information, please contact onepress@plos.org.

Kind regards,

Majid Niazkar, Ph.D.

Academic Editor

PLOS One

Additional Editor Comments (optional):

The manuscript addressed the review comments, and it can be accepted for publication. Before publication, the authors should justify the authorship change at the very last round of the review process.

Reviewers' comments:

# Editor report: Acceptance letter

**DOI:** 10.1371/journal.pone.0354913.r010

**Authors:** Majid Niazkar

PONE-D-25-29739R4

PLOS One

Dear Dr. Shomre,

I'm pleased to inform you that your manuscript has been deemed suitable for publication in PLOS One. Congratulations! Your manuscript is now being handed over to our production team.

At this stage, our production department will prepare your paper for publication. This includes ensuring the following:

* All references, tables, and figures are properly cited

* All relevant supporting information is included in the manuscript submission,

* There are no issues that prevent the paper from being properly typeset

You will receive further instructions from the production team, including instructions on how to review your proof when it is ready. Please keep in mind that we are working through a large volume of accepted articles, so please give us a few days to review your paper and let you know the next and final steps.

Lastly, if your institution or institutions have a press office, please let them know about your upcoming paper now to help maximize its impact. If they'll be preparing press materials, please inform our press team within the next 48 hours. Your manuscript will remain under strict press embargo until 2 pm Eastern Time on the date of publication. For more information, please contact onepress@plos.org.

You will receive an invoice from PLOS for your publication fee after your manuscript has reached the completed accept phase. If you receive an email requesting payment before acceptance or for any other service, this may be a phishing scheme. Learn how to identify phishing emails and protect your accounts at https://explore.plos.org/phishing.

If we can help with anything else, please email us at customercare@plos.org.

Thank you for submitting your work to PLOS ONE and supporting open access.

Kind regards,

PLOS ONE Editorial Office Staff

on behalf of

Dr. Majid Niazkar

Academic Editor

PLOS One
